# Dual role of circulating and mucosal Vδ1 T cells in the control of and contribution to persistent HIV-1 infection

**DOI:** 10.21203/rs.3.rs-4784403/v1

**Published:** 2024-08-02

**Authors:** Brendan T Mann, Marta Sanz, Matthew Clohosey, Kayley Langlands, Alisha Chitrakar, Carles Moreno, Joana Vitalle, Marie Anne Iannone, Ezequiel Ruiz-Mateos, Claire Deleage, Marc Siegel, Natalia Soriano-Sarabia

**Affiliations:** 1. Departments of Microbiology, Immunology and Tropical Medicine and The George Washington University, Washington, DC, USA.; 2. Departments of UNC-HIV Cure Center, Department of Medicine and University of North Carolina at Chapel Hill, Chapel Hill, NC, USA.; 3. Departments of Medicine, School of Medicine and Health Sciences, The George Washington University, Washington, DC, USA.; 4. Institute of Biomedicine of Seville (IBiS), Virgen del Rocio University Hospital, Spanish National Research Council (CSIC), University of Seville, Clinical Unit of Infectious Diseases, Microbiology and Parasitology, Seville, Spain.; 5. Lineberger Comprehensive Cancer Center, University of North Carolina at Chapel Hill, Chapel Hill, NC, USA.; 6. AIDS and Cancer Virus Program, Frederick National Laboratory for Cancer Research, National Cancer Institute, Frederick, MD, USA

## Abstract

Curative strategies for human immunodeficiency virus (HIV-1) infection are hindered by incomplete characterization of the latent reservoir and limited enhancement of anti-HIV immune responses. In this study, we identified a novel dual role for peripheral and tissue-resident Vδ1 T cells within the gastrointestinal mucosa of virally suppressed people with HIV. Phenotypic analyses identified an increased frequency of highly differentiated, cytotoxic effector Vδ1 T cells that exerted potent inhibition of HIV-1 replication *in vitro* coinciding with direct increases in cytolytic function. Conversely, we detected an enrichment of HIV-1 DNA in tissue-resident CD4+Vδ1 T cells *in situ.* Despite low CD4 expression, we found circulating Vδ1 T cells also contained HIV-1 DNA which was replication-competent. We show that TCR-mediated activation of peripheral Vδ1 T cells induced *de novo* upregulation of CD4 providing a plausible mechanism for increased permissibility to infection. These findings highlight juxtaposing roles for Vδ1 T cells in HIV-1 persistence including significant contribution to tissue reservoirs.

Antiretroviral therapy (ART) does not eliminate HIV-1 from the body due to a latent reservoir of cells containing provirus that maintain a quiescent transcriptional state^1,2^. A portion of the viral reservoir harbors escape mutations that confer resistance to humoral and CD8+ T cell responses leading to viral rebound following the cessation of ART^3,4^. Resting memory CD4+ T cells are considered the main contributor to the viral reservoir as they are the primary target of HIV-1. However, growing evidence suggests additional cell types such as circulating monocytes, tissue-resident macrophages, and other non-CD4+ T cells can harbor infectious provirus^5–8^. Improving our understanding of anti-viral immune responses and characterizing the complete latent reservoir will facilitate strategies for eradicating persistent HIV-1 infection.

Unconventional lymphocytes such as γδ T cells are among the majority of T cells that do not typically express CD4. The two main human γδ T cell subsets, Vδ1 and Vδ2, are distinguished by divergent thymic development, tissue localization, and cognate antigens^9,10^. Vδ1 T cells populate mucosal sites such as the gastrointestinal (GI) tract where they act as critical mediators of tissue homeostasis^11,12^. Conversely, peripheral γδ T cells are primarily comprised of innate-like Vγ9Vδ2 T cells with minor frequencies of Vδ1 T cells that have adaptive qualities^10,13,14^. Peripheral Vδ1 T cells expand in response to a limited set of antigens including HIV-1^15^. During early infection, the ratio of Vδ1:Vδ2 frequencies is inverted within the periphery and further pronounced within the GI tract^8,16–18^. Reports of the potential role of Vδ51 T cells in controlling viral replication in the absence of ART suggest they are a critical mediator of anti-HIV immunity^19,20^. Vδ1 T cells can directly lyse or inhibit HIV replication through germline-encoded receptors generally associated with Natural Killer (NK) cells^21,22^. Whether the anti-HIV functions of Vδ1 T cell persist under viral suppression is unclear.

Few studies have investigated the susceptibility of γδ T cells to HIV-1 infection. Nevertheless, both Vδ1 and Vδ2 clones are amenable to infection *in vitro* and HIV-1 provirus has been quantified from isolated pan-γδ T cells by us and others^23–25^. In addition, our group identified peripheral Vδ2 T cells as a cellular reservoir capable of producing replication-competent virus upon reactivation^8^. We further demonstrated that activation induced upregulation of CD4 expression on Vδ2 T cells directly increasing permissibility to infection. These observations indicate that γδ T cells are not only susceptible to HIV-1 infection *in vivo* but may make a meaningful contribution to viral persistence through poorly defined mechanisms. Whether the more abundant Vδ1 subset is targeted by HIV-1 either in circulation or the GI tract remains to be elucidated.

In the present study, we found that circulating Vδ1 T cells from virally suppressed people living with HIV-1 (PWH) on stable ART displayed signatures of continuous activation marked by skewing towards a TEMRA-like (CD45RA+CD27-) phenotype with higher co-expression of cytotoxic markers. Functionally, these Vδ1 T cells retained their proliferative capacity to TCR stimulation and inhibited viral replication *in vitro.* Paired analysis of GI tract revealed a comparable phenotype between both compartments with a few key exceptions including a higher frequency of Vδ1 T cells expressing CD4 within the lamina propria. For the first time, using HIV DNAscope we found tissue-resident CD4+ Vδ1 T cells contain HIV DNA and constitute a previously unrecognized cellular reservoir representing around 20% of all infected CD4+ cells within the GI tract. In an independent cohort containing elite controllers (EC), HIV DNA was also quantified in Vδ1 T cells from the small and large intestine where they comprised 19% of all virally infected CD4+ cells but only 5% in EC. Despite the low *ex vivo* CD4 expression on circulating Vδ1 T cells, replication-competent virus was recovered from 5/11 ART-suppressed PWH. Finally, infectivity and modulation of CD4 expression on Vδ1 T cells from HIV-seronegative donors revealed *de novo* expression of CD4 following TCR stimulation, indicating a potential window of opportunity of increased permissibility to HIV-1 infection. These findings reveal a unique, juxtaposing role for Vδ1 T cells in HIV-1 pathogenesis with direct implications in viral persistence and cure strategies.

## Results

### Participant Characteristics

The ART-suppressed PWH cohorts recruited at US sites were mostly male (91.3%) with a median age of 36 years old. The Spanish cohort was primarily male (100% ART PWH, 66% EC) with a median age of 40. All participants had been on ART for a minimum of 1 year (Table 1). The median age of the HIV- control group was 39 years and also composed of mostly male donors (59.3%).

### Vδ1 T cells display a highly differentiated, cytotoxic phenotype during ART suppression

**Extended data Figure 1:**
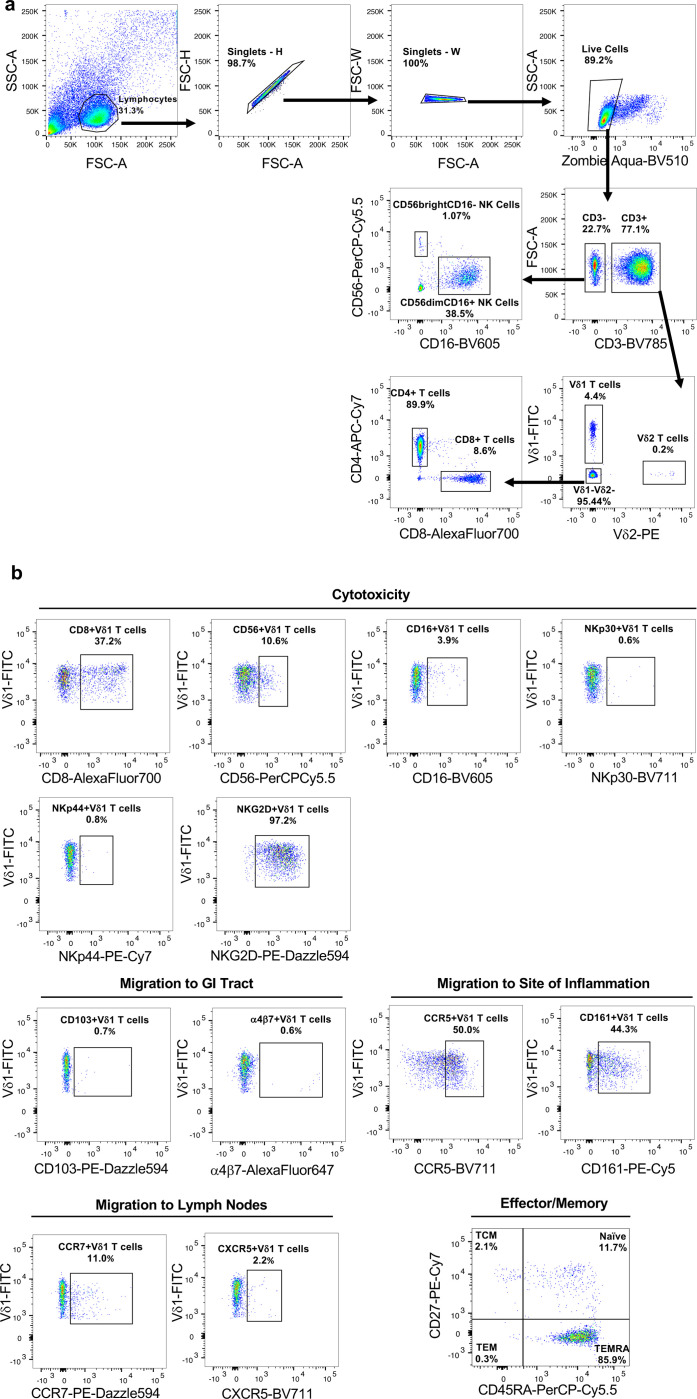
Gating strategy for Immunophenotyping. **a**, Gating strategy defining major γδ T cell (Vδ1 and Vδ2), ab T cell, and NK cell subsets in PBMCs. **b**, Representative pseudocolor plots of marks associated with cytotoxicity, tissue homing, and memory expressed on Vδ1 T cells.

**Extended data Figure 2:**
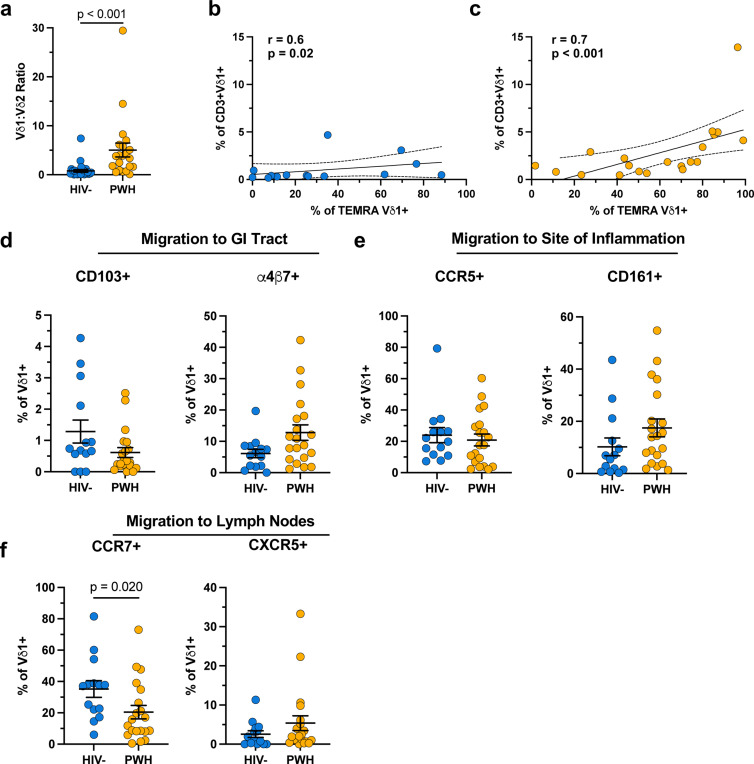
Phenotyping of Vδ1 T cells from HIV- and ART-suppressed PWH. **a**, Vδ1:Vδ2 T cell ratio in HIV- (blue) and ART-suppressed PWH (orange). Spearman’s ranked correlation between the total frequency of circulating Vδ1 T cells and frequency of Vδ1 T cells with a TEMRA (CD45RA+CD27-) phenotype in **b**, HIV- and **c**, ART-suppressed PWH. Frequency of Vδ1 T cells expressing tissue homing markers for the **d**, GI tract, **e**, sites of inflammation, and **f**, lymph nodes within HIV- (blue) and ART-suppressed PWH (orange). Mann-Whitney U test (**a, d-f**). Spearman’s correlation test (**b,c**). Mean ± S.E.M are represented in phenotyping comparisons.

**Extended data Figure 3:**
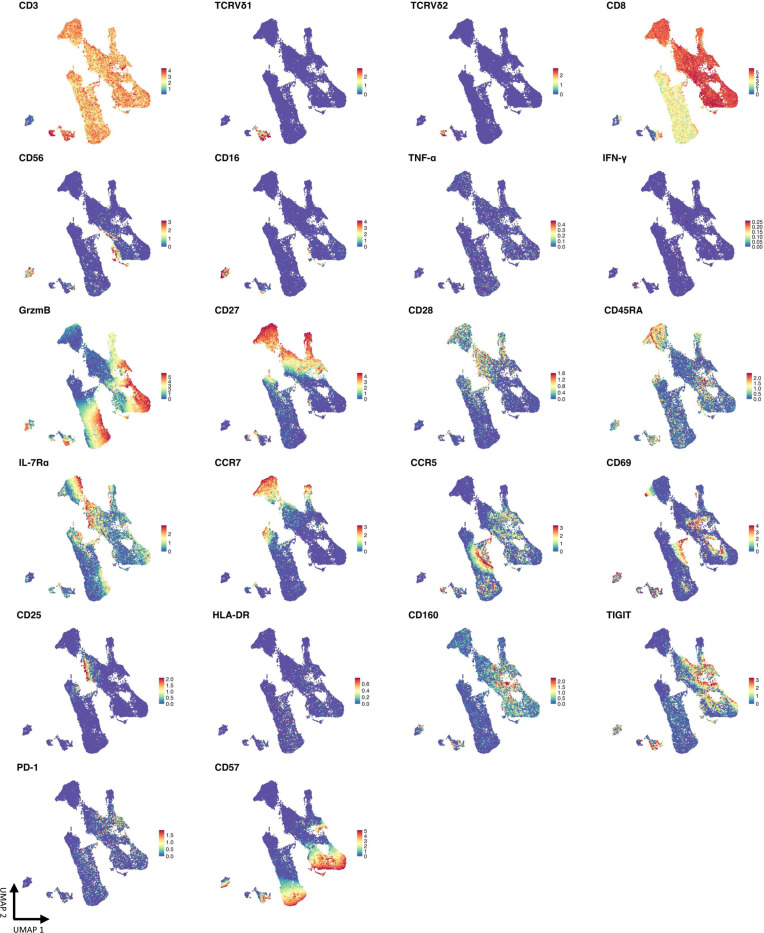
Relative expression of markers associated with cytotoxicity, effector/memory, and activation/exhaustion within FlowSOM clusters. Heatmap of the relative expression of each marker from mass cytometry data overlayed with UMAP displaying clusters identified by FlowSOM analysis in Fig. 1i,j.

**Figure 1:**
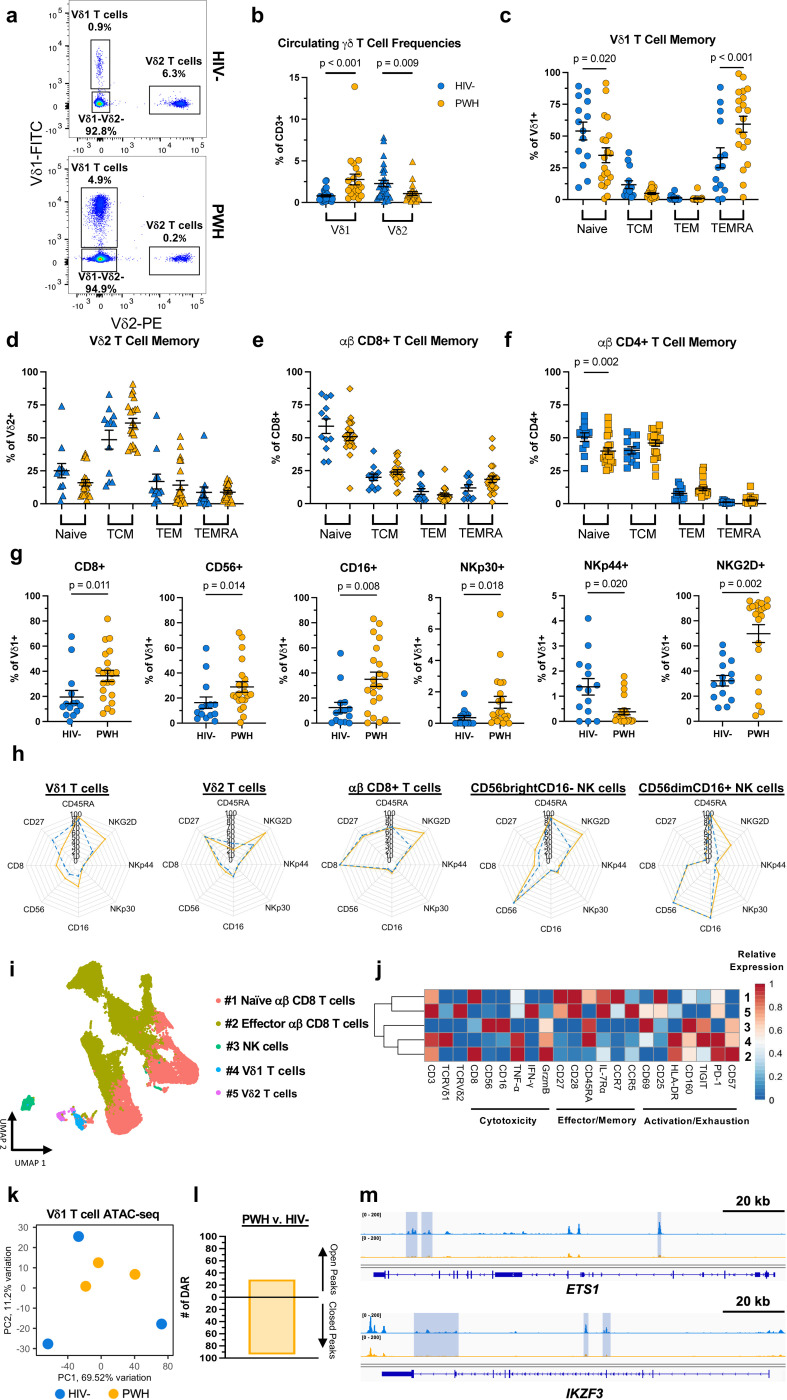
Vδ1 T cells display a highly differentiated, cytotoxic phenotype during prolonged viral suppression. **a**, Representative flow cytometry pseudocolor plots and **b**, frequency of total circulating CD3+ Vδ1 and Vδ2 T cells in HIV- donors (blue, n = 28) and ART-suppressed PWH (orange, n = 21). Frequency of effector/memory subsets within **c**, Vδ1 T cells, **d**, Vδ2 T cells, **e**, ab CD8 T cells, and **f**, ab CD4 T cells in HIV- (n = 14) and PWH (n = 21). **g**, Comparison of cytotoxic marker expression on Vδ1 T cells between HIV- donors (n = 14) and PWH (n = 21). **h**, Radar plots displaying cytotoxic and memory marker expression between different effector cell subsets in HIV- donors (blue, dotted line) and PWH (solid, orange line) from left to right: Vδ1T cells, Vd2 T cells, ab CD8+ T cells, CD56dimCD16 NK cells, and CD56brightCD16- NK cells. **i**, UMAP visualization of effector cell clusters identified by FlowSOM analysis of mass cytometry data from PWH (n = 15) and **j**, heatmap of relative expression of markers associated with cytotoxic function, effector/memory status, and activation/exhaustion within each cluster. **k**, Principal component analysis of ATAC-seq data from purified circulating Vδ1 T cells from HIV- (blue, n =3) and PWH (orange, n = 3). **l**, Number of differentially accessible chromatin regions (DAR) in PWH compared to HIV- controls (absolute shrunkenLog2 FC >0.5, FDR-adjusted p value < 0.1). **m**, Integrated genome viewer window showing reduced ATAC-seq peaks (highlighted in blue) within the *ETS1* and *IKZF3* loci identified from DAR analysis. Mann-Whitney U test (**b,g**), one-way ANOVA using the Holm-Šidák method for multiple comparisons (**c-f**). Mean ± S.E.M are represented.

The expansion of Vδ1 T cell frequencies is maintained throughout suppressive ART^17,26^. In other chronic viral infections such as human cytomegalovirus (CMV), expanded Vδ1 T cells display phenotypic characteristics that resemble adaptive immunity^27,28^. Therefore, we hypothesized that Vδ1 T cells in ART-suppressed PWH possessed phenotypes indicative of enhanced cytotoxicity or tissue homing function. We assessed the expression of memory, cytotoxicity, and tissue homing markers in PWH and HIV-seronegative (HIV-) controls (Extended data Fig 1a,b). Similar to previous reports from us and others^25,26,29^, peripheral Vδ1 T cells from ART-suppressed PWH were expanded compared to HIV- controls (Fig.1a,b) and displayed an inverted Vδ1:Vδ2 ratio (Extended data Fig 2a). In addition, Vδ1 T cells from PWH skewed from a naïve (CD45RA+ CD27+) towards a TEMRA-like (CD45RA+CD27-) phenotype (Fig 1c) which positively correlated with cell frequency in both groups (Extended Data 2b,c). The shift in memory phenotypes was only observed in Vδ1 T cells from PWH whereas Vδ2 T cells and conventional αβ T cells were largely similar between each group apart from lower naïve CD4 T cells in PWH (Fig. 1d-f). In addition, Vδ1 T cells from PWH had increased expression of cytotoxic markers (CD8, CD56) as well as those involved in the recognition or lysis of HIV-infected cells (CD16, NKp30, NKG2D) (Fig 1g). This alteration in effector phenotypes was specific to Vδ1 T cells compared to other cytotoxic cells such as Vδ2 T cells, αβ CD8+ T cells, and NK cells except for higher NKG2D expression which was present in each cell type (Fig 1h). Excluding CCR7 that was less expressed in PWH, receptors or adhesion molecules that mediate lymphocyte migration were distributed similarly in Vδ1 T cells from PWH and HIV- controls (Extended data Fig 2d-f). To confirm persistent HIV-1 infection has a specific signature on Vδ1 T cells, we conducted a deeper phenotypic analysis in 15 PWH using a mass cytometry (CyTOF) panel previously designed by our group^30^. Following FlowSOM analysis, Vδ1 T cells formed a distinct cluster most closely resembling highly differentiated αβ CD8+ T cells (CD27-CD28-CD57+) with both cell types showing higher relative expression of TNF -α and granzyme B (GzmB) compared to Vδ2 T cells and NK cells (Fig 1i,j, Extended Data Fig 3). In addition to displaying a TEMRA-like phenotype, Vδ1 T cells had elevated expression of CD160, TIGIT, and PD-1 indicative of *in vivo* activation occurring during the course of HIV-1 infection (Fig 1j).

**Extended data Figure 4:**
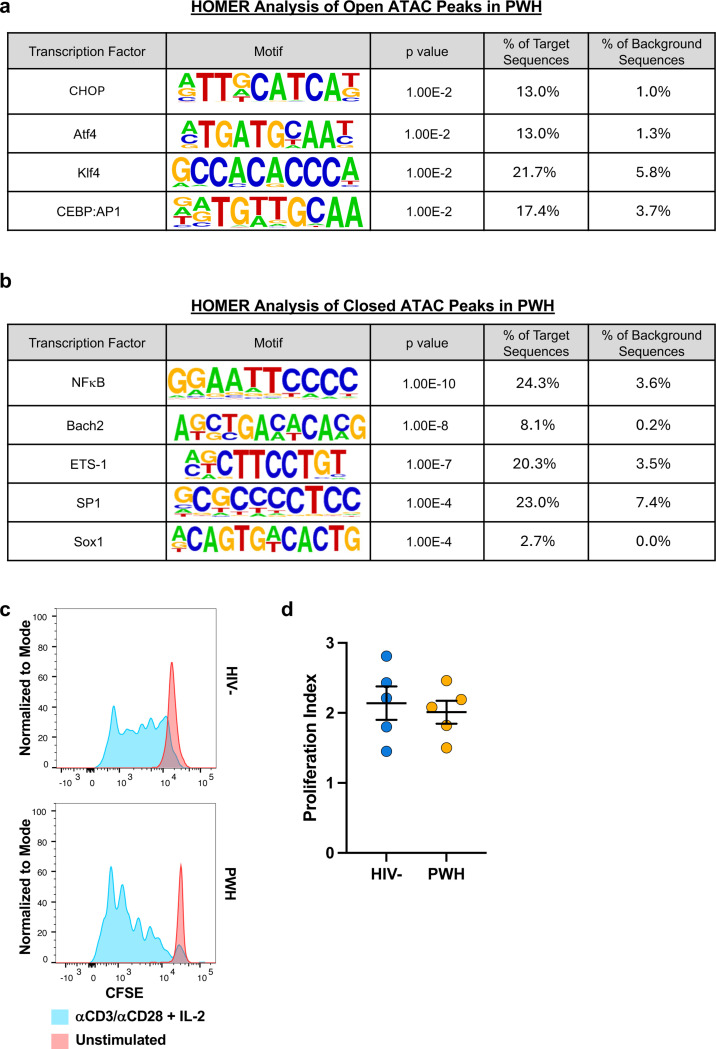
Changes in Transcription factors (TFs) accessibility but not proliferative responses in Vδ1 T cells from ART PWH. TFs with known DNA binding motifs identified by HOMER motif analysis in either **a**, open or **b**, closed chromatin regions of Vδ1 T cells from ART-suppressed PWH compared to HIV-controls. **c**, Proliferative responses of Vδ1 T cells from both HIV- donors and PWH assessed by CFSE following seven days of stimulation with αCD3/αCD28 + IL-2 (blue) compared to unstimulated (red) controls. **d**, Proliferation index of Vδ1 T cells quantified as the number of divisions divided by the number of cells undergoing division. Mean ± S.E.M.

Pathogen-driven Vδ1 T cell activation results in chromatin remodeling which influences subsequent responses to antigen or cytokine production^28^. Therefore, we performed ATAC-seq analysis on purified circulating Vδ1 T cells that revealed only modest differences between PWH and HIV- controls, but a noticeable reduction in interpersonal variation (Fig 1k). Amongst the 123 differentially accessible regions in PWH (29 open, 94 closed) (Fig 1l), the most pronounced repressive changes occurred in regulatory regions or genes that are typically downregulated in HIV-specific effector αβ CD8+ T cells (*IL7Rα, ETS-1*)^31,32^, but also genes that regulate functional T cell exhaustion (*IKZF3, ITPKB, CD5, CD6, CD96*) (Fig 1m, **Supplementary Table 1**). Among the repressed regions was *NCR2* (NKp44), which potentially explains the observed downregulation at the cell surface in ART-suppressed PWH by flow cytometry analysis (Fig 1g). Subsequent HOMER motif analysis identified transcription factor binding sites for Nuclear factor kappa B (NF-κB) in 24.3% of repressed sequences whereas 21.7% of opened regions contain sites for Krüppel-like factor 4 (Klf4) (Extended data Fig 4a,b) which enhances cytotoxicity in exhausted αβ CD8+ T cells^33^. Despite bearing some phenotypic and epigenetic resemblances to exhausted αβ CD8+ T cells, we found Vδ1 T cells from PWH have comparable proliferative responses to HIV- controls following TCR stimulation (Extended data 4c,d). Together these data demonstrate that Vδ1 T cells in PWH display hallmarks of *in vivo* activation and differentiation into cytotoxic effectors despite durable viral suppression.

### Vδ1 T cells from ART-suppressed PWH retain their anti-HIV functionality

**Extended data Figure 5:**
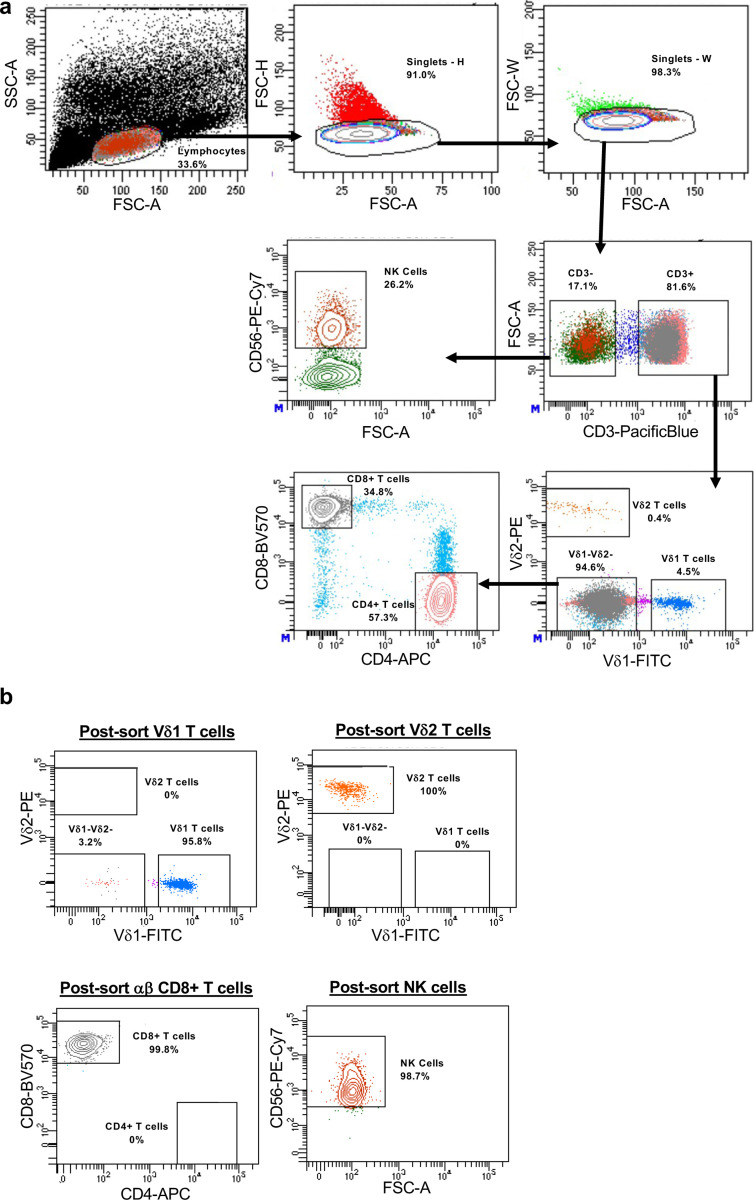
Representative examples of sorting data for viral inhibition assays. **a**, Pre-sort strategy for effector cell populations used in viral inhibition assays shown in Fig 2g. **b**, Post-sort representative pseudocolor plots of each effector cell type.

**Figure 2:**
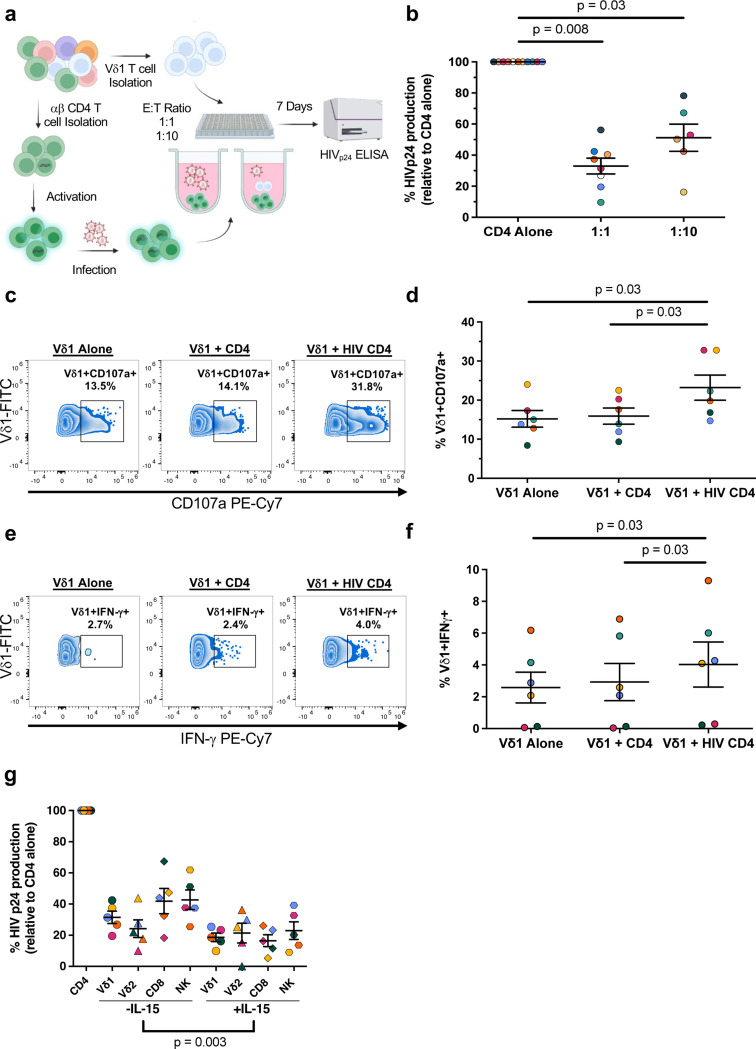
Vδ1 T cells from ART-suppressed PWH retain their anti-HIV capabilities *in vitro.* **a**, Schematic representation of the viral inhibition assay (VIA). ab CD4+ cells from ART-suppressed PWH were superinfected HIV-_JRCSF_ prior to co-culture with autologous Vδ1 T cells for 7 days. **b**, HIV_p24_ production from day 7 supernatants in co-cultures at a 1:1 (n = 8) and 1:10 (n = 6) E:T ratio normalized to ab CD4 T cells cultured alone. Cytotoxicity assay showing **c**, representative zebra plots and **d**, quantification of CD107a expression and **e, f**, intracellular IFN-γ production within Vδ1 T cells cultured alone, co-cultured with non-superinfected ab CD4+ T cells, or with superinfected ab CD4 T cells at a 1:1 ratio (n = 6). **g**, Inhibition of viral replication from super-infected CD4+ T cells by Vδ1 T cells, Vδ2 T cells, ab CD8 T cells, and NK cells with or without pretreatment with IL-15 (n = 5). Friedman test with Dunn’s correction for multiple comparisons (**b,d,f,g**). Means ± S.E.M are represented.

Whether Vδ1 T cell effector functions are maintained during suppressive ART is unclear. To assess anti-HIV function, we performed viral inhibition assays (VIA) previously adapted by our group (Fig 2a, Extended data Fig 5)^34,35^. Co-culture of super-infected autologous αβ CD4+ T cells with purified Vδ1 T cells resulted in a reduction in HIV_p24_ production in a dose dependent manner (mean reduction of 47.9% and 67% at effector:target (E:T) ratios of 1:1 and 1:10, respectively, Fig 2b). Vδ1 T cells specifically degranulated in the presence of super-infected αβ CD4+ T cells compared to co-culture conditions with resting autologous αβ CD4+ T cells (Fig 2c, d). This coincided with a significant increase in the frequency of IFN-γ producing Vδ1 T cells (Fig 2e, f), indicating direct recognition of cells actively producing virus.

To date, efforts to boost anti-HIV cellular responses against the latent reservoir have primarily focused on αβ CD8+ T cells or NK cells^35,36^. This includes the use of pleiotropic compounds such as the IL-15 superagonist N-803 which both reactivates viral transcription and activates cytotoxic effector cells^37^. To assess whether IL-15 may also enhance Vδ1 T cells, we pre-treated the cells prior to the VIA and compared the effects to matching FACS-isolated Vδ2 T cells, αβ CD8+ T cells, and NK cells. A groupwise comparison revealed IL-15 enhanced viral inhibition across all effector cell types (p = 0.003, Fig 2g, Extended data Fig 5). Specifically, pretreatment of Vδ1 T cells with IL-15 led to a greater reduction in HIV_p24_ production compared to untreated effector cells (mean reduction 81.4% v. 68.5% respectively). IL-15 treatment had a comparable effect on matching αβ CD8+ T cells and NK cells, but no discernible impact on Vδ2 T cells. Collectively these experiments demonstrate that not only do Vδ1 T cells retain their anti-HIV cytotoxicity during ART, but also this activity may be enhanced by clinically relevant compounds being pursued for HIV-1 cure.

### Circulating and Vδ1 T cells from the GI are phenotypically distinct with a higher frequency of CD4 expression in the lamina propria

**Extended data Figure 6:**
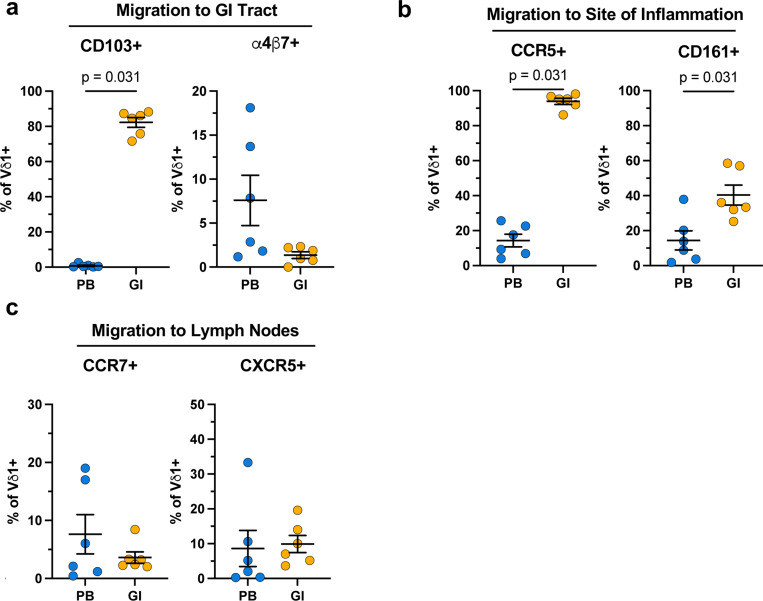
Extended phenotyping of circulating and mucosal Vδ1 T cells from ART-suppressed PWH. **a**, Quantification of tissue homing markers toward the GI mucosa, **b**, sites of inflammation, and **c**, lymph nodes on matched circulating (blue) and GI resident (orange) Vδ1 T cells from ART-suppressed PWH (n = 6). Wilcoxon matched pairs signed rank test. Mean ± S.E.M are represented.

**Figure 3:**
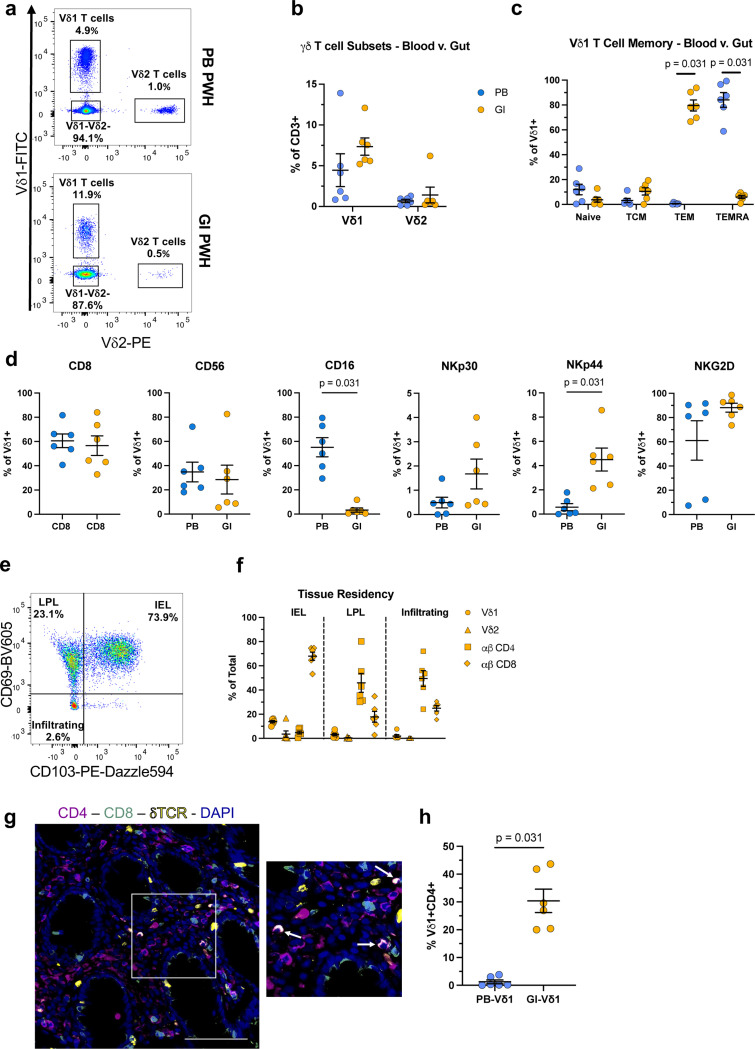
Circulating and mucosal Vδ1 T cells from ART-suppressed PWH have a phenotypically similar cytotoxic profile. **a**, Representative pseudocolor plots and **b**, Vδ1 and Vδ2 T cell frequency, **c**, effector/memory and **d**, cytotoxic marker expression on Vδ1 T cells in matched samples from peripheral blood (PB, blue) and the GI (orange). **e**, Representative pseudocolor plot and **f**, frequency of Vδ1, Vδ2, ap CD4 and CD8 T cell subsets within the total tissue, tissue residency defined as CD103+CD69 intraepithelial lymphocytes (IEL), CD103-CD69+ lamina propria lymphocytes (LPL), and CD103-CD69-infiltrating lymphocytes. **g**, Representative image of IEL and LP Vδ1 T cells by phenotypic immunofluorescent analysis of CD4 (blue), CD8 (orange), and Vδ1 (purple). White arrows point to CD4+Vδ1+ cells exclusively found in LP. scale bar = 100 μm. **h**, Comparison of CD4 expression in Vδ1 T cells between matched PB and GI. Wilcoxon matched pairs signed rank test. (**c,d,i**) Means ± S.E.M are represented (**b-d,f,i**).

Peripheral and tissue-resident Vδ1 T cells possess distinct TCR repertoires and phenotypes likely indicative of their unique roles in immunity^9,38^. How persistent HIV-1 infection impacts this tissue compartmentalization remains poorly understood^18,19^. Therefore, we conducted paired phenotypic analyses comparing circulating and Vδ1 T cells from rectosigmoid biopsies from six ART PWH. Confirming previous studies^18,39^, we found higher frequencies of Vδ1 T cells within the tissue compared to peripheral blood (GI mean: 7.4% v. PB mean: 4.5%, Fig 3a,b). While peripheral Vδ1 T cells were defined by their TEMRA-like phenotype (Fig 1c), their mucosal counterparts displayed a TEM (CD45RA-CD27-) profile (Fig 3c). The expression of select cytotoxic markers was similar between compartments apart from lower CD16 and higher NKp44 within the tissue (Fig 3d). Mucosal Vδ1 T cells also displayed near ubiquitous expression of CCR5 and elevated frequencies of CD161 (Extended data Fig 6b).

We next investigated the tissue-residency status of Vδ1 T cells compared to other lymphocyte populations based on co-expression of the αE integrin (CD103) and CD69 (Fig 3e). Intraepithelial lymphocytes (IEL) defined as CD103+CD69+ were primarily αβ CD8+ T cells (mean 67.9%) followed by Vδ1 T cells (mean 13.9%) with minimal frequencies of other T cell subsets (Fig 3f). By contrast, CD103-CD69+ lymphocytes within the lamina propria (LP) and infiltrating (CD103-CD69-) cells were vastly comprised of αβ T cells with CD4+ cells representing nearly half of each population (mean 45.9% and 49.5%, respectively). Localization of Vδ1, CD4, and CD8 expressing cells within each layer of the mucosa was confirmed by conventional immunofluorescent staining of paraffin-embedded tissue samples (Fig 3g). Our results show that mucosal Vδ1 T cells are phenotypically distinct from those in circulation with divergent patterns of immunological memory and key cytotoxic receptors. Interestingly, *in situ* phenotyping revealed a high proportion of Vδ1 T cells expressing CD4 that localized within the lamina propria (mean 30.4%), in contrast to Vδ1 T cells in PB which were largely CD4- (mean 1.3%, Fig 3h). Raising the question as to whether they can be potential cellular target for HIV-1 and contribute to the latent reservoir.

### Tissue-resident CD4+Vδ1 T cells harbor HIV-1 DNA

**Figure 4:**
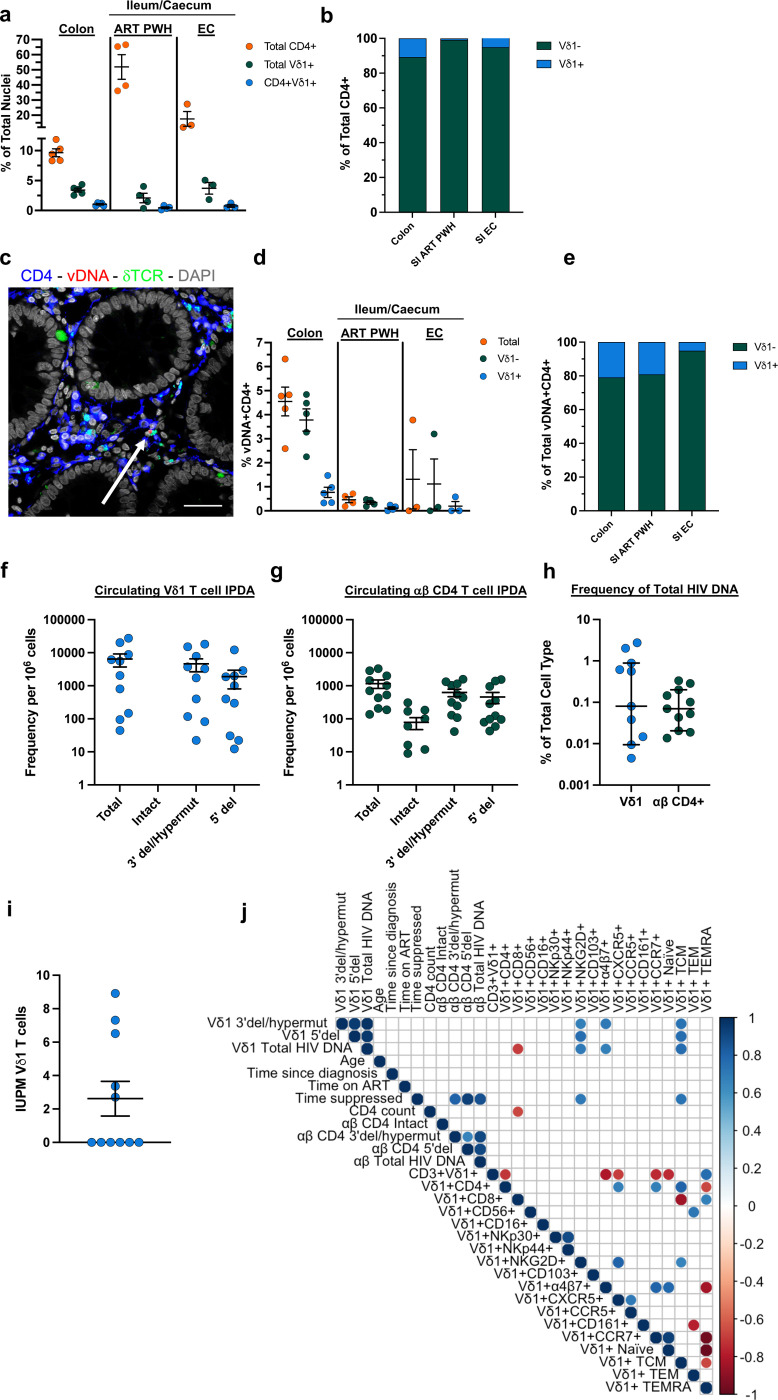
Mucosal and peripheral Vδ1 T Cells are latent reservoirs of HIV-1 in ART-suppressed PWH. **a**, Quantification of CD4+ cells, Vδ1 T cells, and CD4+Vδ1+ T cells relative to total nuclei within the colon of ART-suppressed PWH (GW cohort, n = 5), ileum/caecum of ART-suppressed PWH (SV cohort, n = 4), and ileum/caecum of elite controllers (EC SV cohort, n = 3). **b**, Stacked bar plots of CD4+Vδ1 T cells as a proportion of total CD4+ cells within each section of the GI. **c**, Representative DNAscope image showing HIV-1 viral DNA (vDNA, red) combined with phenotypic immunofluorescent analysis of CD4 (blue) and Vδ1 (green). Arrow is pointing to one infected CD4+Vδ1 T cell that contains vDNA. Scale bar = 100 μm. **d**, Total vDNA+CD4+ cells (orange), Vδ1-CD4+ (green) and Vδ1+CD4+ (blue) cells in the different GI compartments within two independent cohorts of ART-suppressed PWH and ECs. **e**, Stacked bar plots representing the proportion of Vδ1+ and Vδ1-cells amongst total vDNA+CD4+ cells. Frequency of total, intact and defective HIV DNA/10^6^ cells in **f**, purified, circulating Vδ1 T cells and **g**, matching ap CD4 T cells by the Intact proviral DNA Assay (IPDA). **h**, Frequency of infected Vδ1 and αβ CD4+ T cells based on the proportion of cells containing total HIV DNA. **i**, Infectious units per million (IUPM) Vδ1 T cells represent replication-competent virus recovered in quantitative viral outgrowth assays (QVOA). **j**, Correlation matrix between HIV DNA species measured by IPDA, participant clinical characteristics, and phenotype of circulating Vδ1 T cells. Spearman’s ranked correlation test.(**j**). Mean ± S.E.M. (**d, f-i**).

To quantify HIV-1 DNA in tissue-resident Vδ1 T cells, we combined immunofluorescent phenotyping with the ultra-sensitive next-generation HIV DNAScope assay^40^. We used rectosigmoid colon biopsies from ART-suppressed PWH (Fig 3) or ileum and caecum samples from an independent cohort that included ART-suppressed PWH and EC (Table 1). Quantitative image analysis showed CD4+ cells comprised a mean of 9.6% of all cells relative to the total number of nuclei in the colon compared to 51.9% in the ileum/caecum of ART-suppressed PWH and 17.6% in EC. The frequency of Vδ1 T cells expressing CD4 was higher within the colon compared to the ileum/caecum of ART-suppressed PWH (mean 30.3% v. 23.8% respectively, p = 0.02), while the frequency in the ileum/caecum of EC was comparable to ART-suppressed donors (mean 22.6% v. 23.8 Fig 4a). The proportion of CD4+Vδ1 T cells to total CD4+ cells varied between each group with 10.8% in the colon and 0.9% in the ileum/caecum of ART-suppressed PWH, and 5.1% in EC (Fig 4b).

HIV-1 DNA was detected in all ART-suppressed participants within both sections of the GI tract (Fig 4c,d). The frequency of total CD4+ cells containing HIV-1 DNA was 4.6% in the colon and 0.5% in the ileum/caecum of ART-suppressed PWH. In EC, the frequency of HIV infection within total CD4+ T cells was 1.3%. HIV-1 DNA was quantified in Vδ1 T cells in 100% (5/5) of colon samples, 80% of ileum/caecum samples from ART-suppressed PWH, and 33% (1/3) of EC. HIV-1 DNA was exclusively found within Vδ1 T cells expressing the CD4 receptor which represented approximately 20.4% of all HIV-1 DNA+CD4+ cells within the colon and 19.2% in the ileum/caecum of ART-suppressed PWH (Fig 4e). This was contrasted by a lower proportion of infected CD4+Vδ1 T cells in EC (mean 5.1%, Fig 4e). Our results indicate that not only do CD4+Vδ1 T cells contain HIV-1 DNA, but they may be significant contributors to the latent HIV-1 reservoir across the GI tract.

### Peripheral Vδ1 T cells are a latent reservoir of HIV-1

**Extended data Figure 7:**
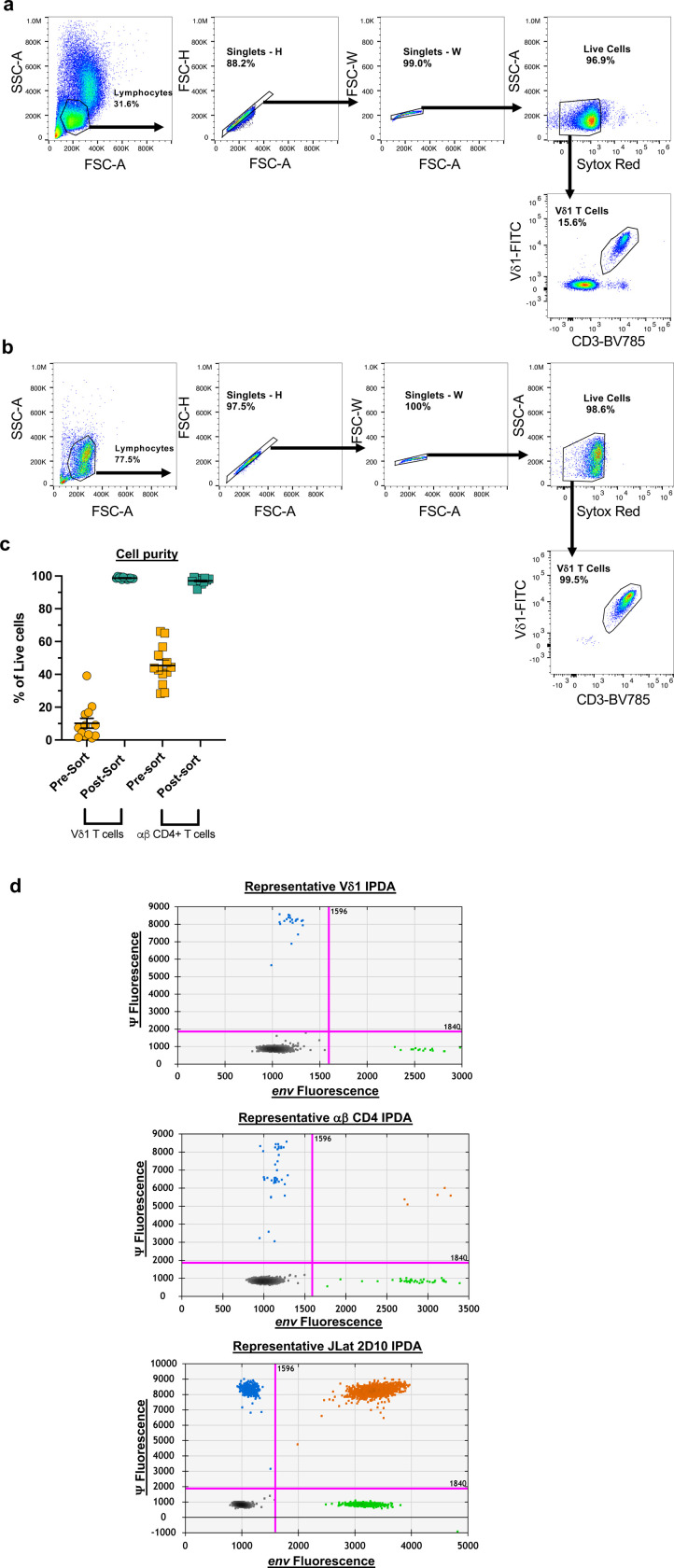
Sorting strategy and representative plots for IPDA. **a**, Sorting strategy for circulating Vδ1 T cells used in IPDA and QVOA (Fig 4). **b**, Representative pseudocolor plots of post-sort purity check on Vδ1 T cells and **c**, Purity of sorted Vδ1 and matched αβ CD4+ T cells. **d**, Representative 2D plots of IPDA conducted on Vδ1 T cells, αβ CD4+ T cells, and JLat 2D10 positive control.

To assess whether Vδ1 T cells contribute to the peripheral latent reservoir, we quantified HIV-1 DNA within FACS-purified Vδ1 T cells by the IPDA (Extended data Fig 7a-d). We detected total HIV-1 DNA in Vδ1 T cells from 91% (10/11) donors including 80% (4/5 donors) of participants with positive HIV DNAscope data (Fig 4f). Defective provirus was detected in each positive sample (**Supplementary Table 2**) with a lower proportion of 5’ defective provirus compared to 3’defective or hypermutated sequences. Intact provirus was not detected within Vδ1 T cells likely due to the limited number of available cells (mean of 44,350 Vδ1 cells/assay, Extended data Fig 7d). By contrast, total HIV-1 DNA in matched αβ CD4+ T cells run in parallel was quantified in all participants (11/11 donors) (Fig 4g) and intact provirus within 73% (8/11) of participants along with both 5’ defective and 3’ defective/hypermutated provirus. Despite the absence of intact provirus, we found a comparably low frequency of circulating Vδ1 T cells and αβ CD4+ T cells contain total HIV-1 DNA (Fig 4h). The IPDA requires additional processing of purified cells to isolate genomic DNA which may result in shearing intact HIV-1 genomes. This presents a unique challenge when quantifying provirus from the limited number of Vδ1 T cells available from small volume blood draws. To overcome these limitations, we conducted quantitative viral outgrowth assays (QVOA) using leukapheresis from additional 11 ART-suppressed PWH (Table 1), as we previously described^8^. A mean of 498,182 absolute circulating Vδ1 T cells were assayed (**Supplementary Table 3**) and infectious virus was recovered in 45% (5/11) of participants with a mean of 2.4 infectious units/10^6^ cells (Fig 4i). These results demonstrate that not only do peripheral Vδ1 T cells contribute to the latent reservoir, but also highlight additional challenges when quantifying latent HIV-1 in rare cell populations.

We next determined whether the frequency of infected Vδ1 T cells was associated with clinical characteristics of the donors (Fig 4j). HIV-1 DNA in Vδ1 T cells was not associated with age, time since diagnosis, time on ART, or CD4 count. We also did not find any associations between HIV-1 DNA copies in matching Vδ1 T cells and αβ CD4+ T cells. Lastly, we sought to identify potential phenotypic markers on Vδ1 T cells associated with viral infection. We found positive correlations between total HIV-1 DNA in Vδ1 T cells and a TCM phenotype as well as with the expression of α4β7, both of which have previously been implicated in the identification of latently infected αβ CD4+ T cells (Fig 4j)^41,42^. Interestingly, there was a strong negative association between the frequency of total HIV-1 DNA in Vδ1 T cells and the frequency of CD8+Vδ1 T cells. Altogether, our findings demonstrate Vδ1 T cells are a novel cellular reservoir of latent HIV-1 infection within the GI mucosa and PB. While our data indicates that CD4 expression is associated with infection of mucosal Vδ1 T cells, how peripheral Vδ1 T cells become infected is still an outstanding question due to the low or absent expression of CD4.

### Infection of Vδ1 T cells is CD4-dependent

**Figure 5:**
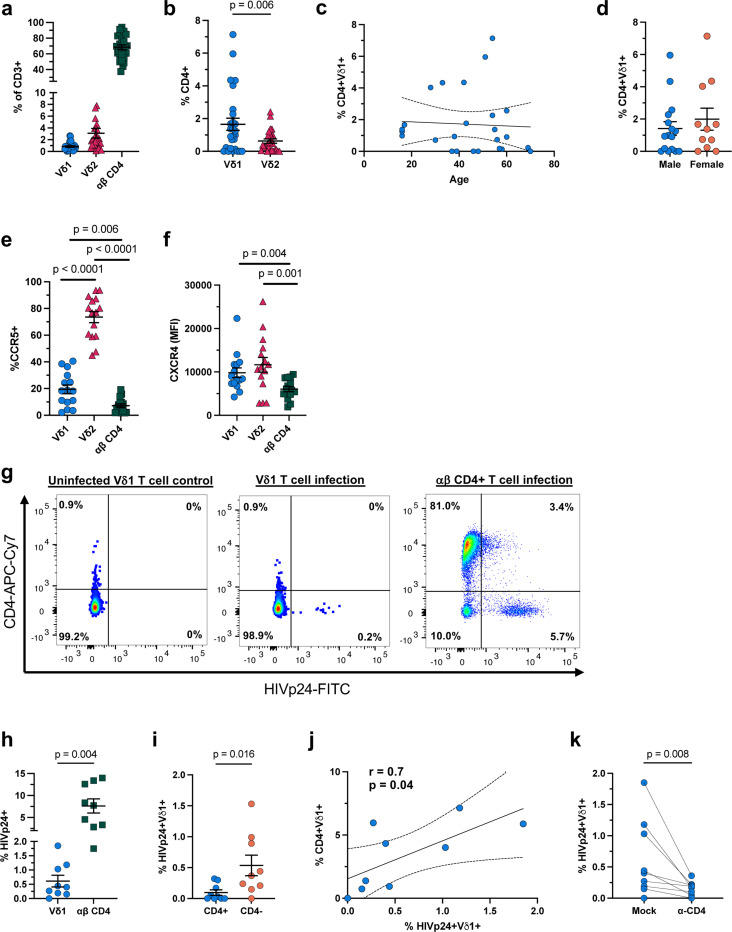
*Ex vivo* infection of Vδ1 T cells from HIV- donors is CD4-dependent. **a**, Frequency of total circulating CD3+ Vδ1, VS2, and αβ CD4+ T cells (n = 27). **b**, Comparison of CD4 expression on Vδ1 and Vδ2 T cells which did not correlate with **c**, age or **d**, biological sex of HIV- donors. Comparison of co-receptors **e**, CCR5 and **f**, CXCR4 expressed on matched Vδ1, Vδ2 and αβ CD4 (n = 15). **g**, Representative pseudocolor plots and **h**, frequency of infected Vδ1 and αβ CD4+ T cells measured as positive for intracellular HIV_p24_ on day 7 of *ex vivo* PBMC infection with HIV_JR-CSF_ (n =9). **i**, Frequency of CD4+ and CD4- Vδ1 T cells positive for HIV_p24_ on day 7 of infection**. j**, Correlation between the frequency of CD4+Vδ1 T cells prior to infection and the frequency of HIV_p24_+ Vδ1 on day 7 of infection. **k**, Comparison of the frequency of HIV_p24_+ Vδ1 T cells with or without αCD4 blockade prior to infection. Wilcoxon matched pairs signed rank test (**b,e,f,h,I,k**). Spearman’s r and p value shown for significant correlations with simple linear regression analyses showing the line of best fit and 95% confidence intervals (**c,j**). Mean ± S.E.M. are represented (**a,b,d-f,h,i**).

We investigated the permissibility of Vδ1 T cells to HIV-1 viral entry *in vitro.* Flow cytometry analysis of HIV-seronegative donors confirmed that circulating γδ T cells are predominantly the Vδ2 subset prior to infection (Fig. 5a). The majority (>90%) of both subsets did not express CD4 *ex vivo* although we found higher frequency and greater variability within Vδ1 T cells (Fig 5b). CD4 expression on Vδ1 T cells was not associated with either the age or self-reported sex (Fig 5c,d). In contrast, both Vδ1 and Vδ2 T cells had significantly higher expression of CCR5 (Fig 5e) and CXCR4 (Fig. 5f) compared to αβ CD4+ T cells. Despite a low frequency of cells expressing CD4, successful infection of Vδ1 T cell clones lacking CD4 expression has been previously reported^23^. To determine the requirement of CD4 for viral entry in primary Vδ1 T cells, PHA-activated PBMCs were exposed to HIV_JR-CSF_ in the presence or absence of a monoclonal antibody blocking CD4 prior to infection. As expected, the proportion of infected Vδ1 T cells was significantly lower than αβ CD4+ T cells (Fig 5g,h). The majority of HIV_p24_+ Vδ1 T cells did not express CD4 (Fig 5i) which is compatible with the downregulation of CD4 induced by the viral accessory proteins vpu and nef^43^. Nonetheless, we observed a positive correlation between CD4 expression on Vδ1 T cells prior to infection and the proportion of infected cells after seven days of infection (Fig 5j) with CD4 blockade successfully abrogating infection (Fig 5k). Together these data demonstrate that the primary mechanism of viral entry and infection of Vδ1 T cells is CD4-dependent.

### TCR stimulation induces CD4 upregulation in a discrete subset of Vδ1 T cells

**Figure 6:**
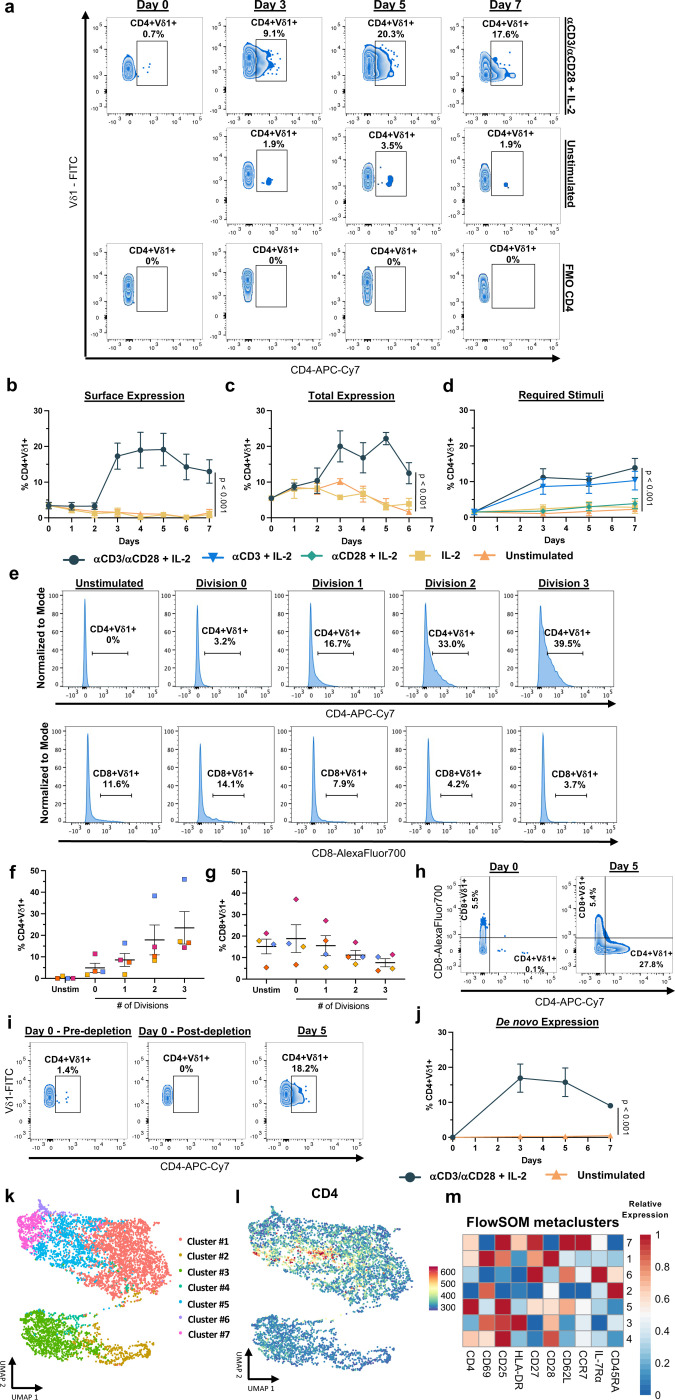
Activation induces upregulation of CD4 expression on circulating Vδ1 T cells from HIV-donors. **a**, Representative zebra plots and quantification of time course **b**, surface and **c**, total CD4 expression on circulating Vδ1 T cells (n = 5) following stimulation with αCD3/αCD28 + IL-2 (navy), IL-2 alone (yellow), or unstimulated (orange). **d**, Comparison of stimuli requirements for CD4 expression on Vδ1 T cells (n = 8) in time course analysis following stimulation with αCD3/αCD28 + IL-2 (navy), αCD3 + IL-2 (blue), αCD28 +IL-2 (green), IL-2 alone (yellow), or unstimulated (orange). **e**, Representative histograms and frequency of **f**, CD4 and **g**, CD8 expressing Vδ1 T cells (n = 4) across cell divisions following 5 days of stimulation with αCD3/αCD28 + IL-2. **h**, Representative zebra plots from day 0 and day 5 following stimulation demonstrating CD4 and CD8 expression are confined to discrete single positive Vδ1 T cell populations. **i**, Representative zebra plots and **j**, frequency of CD4 expression after depletion and subsequent de novo expression on Vδ1 T cells following stimulation with αCD3/αCD28 + IL-2 (n = 4) quantified on day 0, day 3, day 5, and day 7. **k**, UMAP visualization of total Vδ1 T cell clusters identified by FlowSOM analysis of the expression of CD4 and markers associated with activation and differentiation 5 days after stimulation (n = 6). **l**, UMAP of relative CD4 expression overlaid onto FlowSOM clusters and m, heatmap of relative expression of each marker showing a concentration of CD4 expression in cluster #5 and absence of CD4 in cluster #2. Two-way ANOVA with Holm-Šidák method for multiple comparisons (**b-d**,**j**). Mean ± S.E.M. is represented.

Our finding that peripheral Vδ1 T cells contain HIV-1 provirus (Fig 4i) indicates that infection occurs *in vivo* despite low CD4 expression (Fig 3i). Based on the rapid expansion and differentiation of Vδ1 T cells during HIV-1 infection, we investigated the dynamics of CD4 expression following activation in samples from HIV- donors. Within the first seven days following αCD3/αCD28 and IL-2 stimulation, the mean frequency of Vδ1 T cells expressing surface or total CD4 increased from a mean of 3.4% peaking between day three to five (mean 18.2%, Fig 6a-c). The combination of all three stimuli consistently produced the highest frequency of CD4+Vδ1 T cells (Fig 6d). However, neither αCD28 co-stimulation nor IL-2 supplementation alone were sufficient to increase CD4 expression compared to unstimulated controls. Interestingly, CD4 expression increased with each successive division after αCD3/αCD28 + IL-2 stimulation (Fig 6e, 6f) which was contrasted by lower proliferation of CD8+Vδ1 T cells (Fig 6e,g). Our data demonstrates that the induction of CD4 expression in Vδ1 T cells requires TCR-CD3 complex signaling, possibly linking modulation to antigen recognition. However, CD4 expression is excluded from CD8+Vδ1 T cells (Fig 6h) possibly suggesting the existence of unique mechanisms that regulate CD4 expression following activation in a discrete Vδ1 T cell subset.

These results raised the possibility that pre-existing CD4+Vδ1 T cells may have more robust proliferative responses than CD4- cells. Therefore, we depleted CD4+ cells from PBMCs prior to stimulation and analyzed CD4 expression kinetics. Upon activation of Vδ1+CD4- cells, we observed *de novo* CD4 expression in a mean frequency of 15.7% with similar kinetics to activation of whole PBMCs (Fig 6i,j). We next performed ATAC-seq analysis from Vδ1 T cells isolated *ex vivo* revealing that the *Cd4* locus was largely repressed within both seronegative individuals and virally suppressed PWH (Extended data 8). CD4 expression is regulated through multiple epigenetic or transcriptional mechanisms throughout the development of αβ T cells including DNA methylation^44^. Therefore, using a methylation-sensitive restriction enzyme-based assay, we were able to directly detect methylation of CpG dinucleotides within the *Cd4* promoter region of a portion of Vδ1 T cells in both unstimulated and TCR-stimulated conditions (Extended data Fig 9a-c). To determine the role of methylation in CD4 expression, we utilized the DNA methyltransferase I (DNMT1) inhibitor 5-Azacytidine (5-Aza) which prevents the carryover of homeostatic methylation in daughter cells following cell division. Inclusion of 5-Aza in PBMC cultures stimulated with αCD3/αCD28 and IL-2 had a consistent additive effect on the frequency of CD4+Vδ1 T cells five days after activation compared to mock treated controls (Extended data Fig 9 c,d). Together these data shows that a subpopulation of Vδ1 T cells that express CD4 follows discrete epigenetic programs that differentiates it from other subpopulations such as CD8+Vδ1 T cells.

Next, we assessed whether CD4+Vδ1 T cells constitute a unique subpopulation potentially associated with a discrete activation and differentiation profile. Within 5 days of stimulation, high frequencies of Vδ1 T cells expressed CD69, CD25, and HLA-DR showing a spectrum of cells within early, mid, and late-stage activation respectively (Extended Data Fig 10a). While there were minimal changes in co-stimulatory molecules CD27 and CD28, significant proportions of Vδ1 T cells had downregulated naïve markers CD45RA and IL-7 receptor a (IL-7Rα) and upregulated lymphoid homing receptors CD62L and CCR7 (Extended Data Fig 10b-d). To better understand the distribution of CD4 expression within different Vδ1 T cell subsets, we performed FlowSOM analysis. CD4 expression was concentrated in a distinct cluster (cluster #5) featuring the highest relative expression of CD25 and intermediate expression of CD27, CD28, and CD62L with low expression of CD45RA and IL7Rα (Fig. 6k-m, Extended data Fig 10e). On the contrary, CD4-Vδ1 T cells (cluster #2) had the lowest expression of CD25, CD27, CD28, and CD62L along with the highest expression of CD45RA (Fig. 6m). Our findings show that a specific subset of Vδ1 T cells upregulates CD4 following activation and may represent a distinct population that become susceptible to HIV-1 infection *in vivo.*

## Discussion

γδ T cells are not only a central component to immunosurveillance of mucosal tissue, but also play a crucial role in response to microbial infections^45^. HIV-1 infection permanently reshapes the γδ T cell compartment with the most pronounced effects occurring in the Vδ1 subset both within the periphery and the GI tract^18,39^. In the present study, we reveal a novel dichotomy for circulating and mucosal Vδ1 T cell subpopulations with discrete functional contributions in ART-suppressed PWH. A subset of highly differentiated, cytotoxic effectors that strongly inhibits HIV-1 replication and a second, possibly distinct subset expressing the CD4 receptor that makes a meaningful contribution to the latent HIV-1 reservoir.

In agreement with previous studies, we found ART-suppressed PWH had elevated frequencies of peripheral Vδ1 T cells that skewed towards a TEMRA-like phenotype coinciding with higher expression of cytotoxic receptors^19,26^. Although γδ T cells have historically been considered innate-like, recent evidence suggests that certain subsets can undergo clonal expansion and assume properties more closely aligned with adaptive αβ CD8+ T cells responses^13^. Our data revealed a strong positive correlation between the proportion of CD8+Vδ1 T cells and both total circulating frequencies and TEMRA-like Vδ1 T cells suggesting a link between CD8 expression and *in vivo* effector differentiation. The specific expansion of CD8+Vδ1 T cells observed in other diseases such as cardiovascular disease or chronic antigen exposure that occurs in bacterial (*mycobacterium tuberculosis*) or viral (CMV) infections as well as cancer may be a byproduct of prolonged exposure to inflammatory conditions^46^. The proposed etiology of Vδ1 T cell expansion in PWH has ranged from recognition of gut-derived microbial antigens to HIV-mediated impairment of tissue homing, but a definitive mechanism has yet to be described^47,48^. IL-7 signaling was recently implicated as the main driver behind the expansion of thymic derived CD8+Vδ1 T cells^49^. Plasma IL-7 levels are elevated in untreated HIV-1 infection and subsequently restored along with IL-7Rα expression on αβ T cells during ART^50^. Instead, we observed persistent downregulation of IL-7Rα on Vδ1 T cells suggesting continuous exposure to antigen may occur despite suppressive ART.

Few studies have investigated the functional consequences to the phenotypic changes in Vδ1 T cells during ART suppression^26^. To our knowledge, our study provides the first comparative analysis of both phenotype and anti-HIV function between matched circulating γδ T cell subsets, αβ CD8+ T cells, and NK cells. We found Vδ1 T cells from ART-suppressed individuals displayed a higher frequency of cells expressing NKp30 and CD16 compared to HIV-controls similar to NK cells. Similarly, both cell types co-expressed CD160 and TIGIT indicating potential shared regulatory mechanisms of their effector functions^29,51^. Despite these similarities to NK cells, unsupervised clustering analysis indicated that Vδ1 T cells are most similar to antigen-experienced αβ CD8+ T cells with the highest relative expression of TNF-α and GzmB. Perhaps more importantly, we demonstrate that circulating Vδ1 T cells potently inhibited HIV-1 replication of superinfected αβ CD4+ T cells. Inhibition coincided with increased degranulation and IFN-γ production suggesting direct contact and lysis of infected target cells. Whether this recognition is mediated through the TCR, a germline-encoded receptor, or combination is an intriguing question. Furthermore, we found Vδ1 T cell anti-HIV function to be enhanced by pretreatment with the γc cytokine IL-15 similar to αβ CD8+ T cells and NK cells. The positive effects of IL-15 on γδ T cell cytotoxicity, and in particular effector Vδ1 T cells, is well established^27,52^. Our data show that Vδ1 T cells in ART-suppressed PWH retain anti-HIV responses which warrants further attention in clinical studies aimed at eradicating the latent HIV-1 reservoir.

A major finding from our study is the high prevalence of CD4+Vδ1 T cells across the GI tract that contained HIV-1 DNA. CD4+Vδ1 T cells constituted up to 30% of all infected CD4+ cells within the colon and the small intestine. Interestingly, HIV-1 DNA was also quantified in only one out of three samples from the GI tract of EC, which is compatible with a reported strong negative correlation between the percentage of pro-inflammatory Vδ1 T cells and viral load within the GI tract of EC^19^. Tissue-resident Vδ1 T cells primarily localize within the epithelium where they are critical mediators of tissue surveillance^9^ or in smaller fractions within the lamina propria where they play a role in crosstalk with commensal microbiota^53^. Both the presence of CD4 expression and HIV-1 DNA were limited to LP Vδ1 T cells whereas IEL Vδ1 T cells displayed similar expression of cytotoxic markers to their circulating counterparts. Our data suggests an opposing role for Vδ1 T cells based on tissue localization during persistent infection.

Lastly, we found that circulating Vδ1 T cells harbor both HIV-1 DNA and replication-competent provirus similar to the Vδ2 subset previously characterized by our group^8^. We quantified HIV-1 DNA within 91% of participants and infectious virus was recovered from 45% of participants indicating a high prevalence of *in vivo* infection despite low frequencies of circulating CD4+Vδ1 T cells. For the first time, we show that the *Cd4* locus is repressed *ex vivo* and that stimulation through the TCR-CD3 complex resulted in *de novo* expression of CD4 on a discrete Vδ1 T cell subset. This subset was distinct from CD8+Vδ1 T cells, highly proliferative, and associated with high CD25 expression which is an indicator of TCR signaling strength^54^. Furthermore, our detection of CpG methylation within the *Cd4* promoter region of only a portion of Vδ1 T cells suggest divergent epigenetic programs exist for cells that upregulate the receptor versus those that do not. These findings define two specific Vδ1 T cell subsets, one that becomes permissible to HIV-1 infection and directly contributes to the latent reservoir and one cytotoxic subset that is potentially resistant to HIV-1 infection.

Our study has several limitations. Our cohorts were primarily male with a narrow range of ages, which may influence γδ T cell frequencies and phenotype as previously reported by us and others^10,55^. Second, our inability to detect intact provirus with the IPDA due to a limited number of cells precludes a more accurate quantification of latently infected Vδ1 T cells. However, the proportion of intact and defective provirus within αβ CD4+ T cells was comparable to previous studies and we did not observe amplification failure which can occur due to interpersonal sequence variation within the viral reservoir^56,57^. Furthermore, while we successfully recovered replication-competent virus from 5/11 donors, the anti-HIV capabilities of Vδ1 T cells may present an additional confounding factor that limits viral outgrowth in a traditional QVOA. Given the rarity of αβ CD4+ T cells with intact provirus (~100 copies per 10^6^ cells)^58^ and even less frequent inducible provirus (~1 per 10^6^ cells)^59^, these findings underscore important considerations for assay selection when attempting to quantify rare cellular reservoirs.

In summary, our study has revealed a previously unknown role for circulating and mucosal Vδ1 T cells in HIV-1 persistence. Unlike other chronic viral infections^27,28,60^, our work highlights the unique immunological circumstance of persistent HIV-1 infection, characterized by Vδ1 T cells that have opposing roles in both controlling and contributing to latent infection. Determining the specificity of these cytotoxic Vδ1 T cells and characterizing the complete contribution of CD4+Vδ1 T cells as a latent reservoir will improve our understanding of the role of γδ T cells in the greater context of anti-viral immunity and could prove fruitful for HIV-1 cure strategies.

## Methods

### Study subjects

Participants were enrolled and written informed consent obtained at the George Washington University, the University of North Carolina, and the Virgen del Rocio University Hospital/Institute of Biomedicine of Seville through IRB approved protocols. Peripheral blood mononuclear cells (PBMCs) were isolated from 53 virally suppressed PWH enrolled at either George Washington University or the University of North Carolina, Chapel Hill (Table 1). Additionally, 3–5mm^3^ biopsy samples were harvested from the rectosigmoid colon within six participants for paired analyses of tissue-resident Vδ1 T cells. Embedded biopsies from both the small and large intestine of an independent cohort including ART-suppressed PWH (3 ileum, 1 caecum) and EC (2 ileum, 1 caecum) enrolled at the Institute of Biomedicine of Seville were included in experiments quantifying HIV DNA within the GI mucosa.

Buffy coats were purchased from the Gulf Coast Regional Blood Center without personal identifiers. PBMCs isolated from 27 HIV-seronegative individuals were used as controls in appropriate experiments.

### Gastrointestinal biopsy tissue preparation

Sixteen 3 – 5 mm^3^ biopsies were obtained via sigmoidoscopy and colonoscopy procedure with single-use, EndoJaw Jumbo forceps with a 3.7 mm channel (Olympus medical) from 10 ART-suppressed PWH and 3 EC. Biopsy specimens were placed in RPMI (GI-RPMI) supplemented with 20% pooled Human AB serum (Innovative Research, Inc.), 2% Penicillin/Streptomycin (Sigma-Aldrich), 250 ng/mL Gentamicin (Gibco), and 5 ng/mL Amphotericin B (Gibco) then placed on ice until further processing. Four whole biopsies from each participant were fixed in 10% neutral buffered formalin at room temperature for 24 h then washed in increasing concentrations of ethanol and cleared with xylene prior to paraffin embedding. The remaining 12 biopsies were rinsed in RPMI, and then the tissue was dissociated using a three-step procedure to isolate cells for phenotyping: (1) Disruption of the epithelial layer: biopsies were placed in 20 mL of media (ER Solution) containing Hanks Buffered Salt Solution (Gibco), 10% fetal bovine serum (FBS), 10 mM EDTA (Invitrogen), and 5 mM DTT (Sigma-Aldrich) then incubated at 37°C for 20 min within an orbital shaker set to 130 rpm. The media was then decanted through a 70 μm cell strainer and biopsies were transferred to a fresh tube containing 20 mL of ER solution for a second round of incubation. The biopsies were then rinsed in 20 mL of RPMI containing 10 mM HEPES (FisherScientific), and 5% FBS and incubated at 37°C for 20 min within an orbital shaker set to 130 rpm. Media from each incubation was pooled and centrifuged at 600 × *g* for 10 min to collect intraepithelial lymphocytes. (2) Digestion of the lamina propria: Following epithelial disruption, biopsies were placed in 1 mL of RPMI with 0.5 mg/mL of collagenase D (Sigma-Aldrich), 0.1 mg/mL DNase I (StemCell Technologies), and 10 mM HEPES (FisherScientific) then incubated at 37°C for 30 min in a water bath. Samples were vortexed every 3–4 min throughout the incubation. (3) Mechanical disruption: Following enzymatic digestion, biopsies were mechanically disrupted through a 70 μm cell strainer using a 16-gauge blunt ended syringe (StemCell Technologies. The strainer was rinsed with 50 mL of GI-RPMI and centrifuged at 600 × *g* for 10 min. The supernatant was aspirated, and cells isolated from both epithelium disruption (IEL fraction) and enzymatic digestion (LP fraction) were combined and incubated in 1 mL RPMI with 0.1 mg/mL DNase I and 10 mM HEPES at room temperature for 30 min. Following the final wash, cells were resuspended in GI-RPMI, counted three times with a LUNA-FL cell counter (Logos Biosystems), and incubated at 37°C overnight prior to phenotyping.

### Flow cytometry immunophenotyping and cell sorting

PBMCs were isolated by Ficoll-gradient, and viably stored in 20% DMSO and FBS in Liquid Nitrogen until further use. Cells were thawed and counted three times with a LUNA-FL cell counter (Logo Biosystems). Phenotyping of PBMCs and dissociated tissue was conducted using a core panel of titrated fluorochrome-conjugated monoclonal antibodies (all from Biolegend unless otherwise stated) targeting lineage defining markers: CD3 (clone SK7), Vδ1 (clone REA117, Miltenyi), Vδ2 (clone B6), CD4 (clone SK3), and CD8 (clone SK1) followed by additional markers split into three separate panels describing immunological memory: CD45RA (clone HI100), CD27 (clone M-T271), CCR7 (clone G043H7), cytotoxicity: CD56 (clone 5.1H11), CD16 (clone 3G8), NKp30 (clone P30–15), NKp44 (clone P44–8), NKG2D (clone 1D11), and tissue homing: CD103 (clone Ber-ACT8), a_4_b_7_ (clone Hu117, R&D Systems), CCR5 (clone J418F1), CD161 (clone DX12, BD Biosciences), and CXCR5 (clone J252D4). Cells were harvested, washed, and stained with a viability dye (Zombie Aqua fixable viability kit, BioLegend) followed by surface staining with each respective panel for 20 min in the dark at 4°C. Cells were then washed and fixed in 2% PFA. Samples were acquired in a LSRFortessa X-20 (BD Biosciences) and analyzed using FlowJo software v10.10.0 (BD Biosciences). Either Vδ1 T cells or other effector cells (Vδ2 T cells, αβ CD8+ T cells, NK cells) were directly sorted from PBMCs (gating strategies shown in Extended data Fig 5 and Extended data Fig 7) using a FACSAria II (BD Biosciences) or SH800 (Sony Biotechnology). PBMCs were depleted of αβ T cells (StemCell Technologies) prior to sorting Vδ1 T cells to enrich the pre-sort sample.

### Mass Cytometry (CyTOF) and clustering and dimensionality reduction analysis

Additional phenotyping was conducted on thirteen ART PWH using a mass cytometry panel and protocol previously published by our group^30^. Briefly, three million PBMCs were stained with a monoclonal antibody targeting CD45 conjugated to non-radioactive yttrium (89Y) and spiked with a single control donor labeled with Indium 115 (115In). Cells were then stained with monoisotopic cisplatin 198 to discriminate viability per manufacturer suggestions (Fluidigm) followed by incubation with antibodies targeting surface markers in cell staining buffer (CSB) for 30 min at room temperature. Cells were then washed twice in CSB, fixed in 2% paraformaldehyde for 60 min, then washed once with Perm/Wash Buffer (Biolegend). Cells were incubated for 30 min at 37°C with the intracellular antibody cocktail in Perm/Wash Buffer. Stained cell suspensions were barcoded (20-Plex Pd Barcoding Kit, Fluidigm), pooled, resuspended in 62.5 nM Cell-ID Intercalator-Ir (Fluidigm) in Maxpar Fix and Perm Buffer (Fluidigm) and incubated overnight at 4°C. Immediately prior to data acquisition, samples were centrifuged, washed in CSB, followed by a wash in Cell Acquisition Solution (CAS, Fluidigm), filtered (40 μm Cell Strainer, FlowMi), and cell concentration adjusted in CAS to 0.5 × 10^6^ cells/mL with addition of 10% (v/v) EQ Four Element Calibration Beads (Fluidigm). Samples were acquired on a Helios mass cytometer (Fluidigm) equipped with a WB injector.

CyTOF data was initially prepared in FlowJo v10.10.0 (BD Biosciences) by gating on events based on singlets, viability, DNA content, and 87Y staining to differentiate samples from spiked in control. Myeloid cell populations (Monocytes, Macrophages, Dendritic cells) and B cells were excluded by gating on events negative for CD14, CD33, CD11c, CD123, and CD19. Effector cell populations were determined based on gating strategies defined in Extended data Fig 1a. The populations of interest from each donor were concatenated and exported as raw value CSV files. Computational analysis of data was performed using the Spectre R package^61^. Values were arcsinh transformed using a co-factor of 10 to redistribute the data on a linear scale and compress low end values near zero. The data was then merged and unbiased hierarchical clustering performed using the FlowSOM algorithm (metacluster k = 5)^62^. Cell types were annotated based on analysis of marker expression within each metacluster. Subsequently, the data was downsampled to 50,000 events and visualized by the Uniform Manifold Approximation and Projection (UMAP).

### ATAC-seq and data analysis

Isolated cryopreserved Vδ1 T cells were sent to Active Motif (Carlsbad, CA) to perform the Fixed Cell ATAC-seq assay. The cells were processed according to the Fixed Cell ATAC-Seq Kit instruction (Active Motif cat# 53151) except for 30 min of cell fixation incubation time with cell fixation buffer. Resulting material was quantified using iSeq (Illumina) and sequenced with PE42 on the NoaSeq6000 (Illumina). Reads were aligned using the BWA algorithm (mem mode; default settings). Duplicate reads were removed, only reads mapping as matched pairs and only uniquely mapped reads (mapping quality ≥ 1) were used for further analysis. Alignments were extended *in silico* at their 3’-ends to a length of 200 bp and assigned to 32-nt bins along the genome. The resulting histograms (genomic “signal maps”) were stored in bigWig files. Peaks were identified using the MACS v2.1.0 algorithm at a cutoff of p-value 1e-7, without control file, and with the -nomodel option. Peaks that were on the ENCODE blacklist of known false ChIP-Seq peaks were removed. Signal maps and peak locations were used as input data to Active Motifs proprietary analysis program. HOMER motif analysis was performed using BED files of differentially accessible peaks as input for the findMotifsGenome.pl command with a 200 bp search area centered around the midpoint of the differential region (+100 bp, −100 bp). The resulting identified motifs were enriched across all sequences whereas individual peak regions are not annotated with specific motifs.

### Generation of HIV-1 viral stock

HEK293T cells (ATCC) were seeded and cultured to 50–80% confluency prior to transfection with Fugene6 (Promega) and HIV_JR-CSF_ plasmid DNA (NIH AIDS Reagent Program) at a ratio of 3:1. Twenty-four hours later, supernatant was removed and replenished with fresh RPMI + 10% fetal bovine serum. After an additional 24 hours, supernatant was harvested and filtered through a 0.45 μm polyethylsulfone membrane to remove cellular debris and frozen at −80°C. Viral titers were quantified by HIV_p24_ ELISA (Perkin Elmer).

### *In vitro* HIV-1 infection of PBMCs and isolated CD4+ T Cells

PBMCs from HIV-seronegative donors were activated with 3 μg/mL PHA and 100 U/mL IL-2 for 48 hours. Cells were then washed twice and infected with the viral strain HIV_JR-CSF_ by spinoculation at 2000 × *g* for 2 hours. Cells were washed twice to remove unbound virus, resuspended in cRPMI supplemented with 100 U/mL IL-2 (Peprotech), and cultured in 96-well plates for 7 days. Infection was confirmed by flow cytometry, cells were harvested, washed, and stained with a viability dye (Zombie Aqua fixable viability kit, BioLegend) followed by surface staining with monoclonal antibodies (Biolegend) against CD3 (clone SK7), Vδ1 (clone REA117, Miltenyi), and CD4 (clone SK3). Cells were then fixed and permeabilized with Cytofix/CytoPerm (BD Biosciences) for 20 min at 4°C in the dark. Cells were then washed in 1X Perm/Wash Buffer (BD Biosciences) and stained for intracellular HIV_p24_ (clone KC-57, Beckman Coulter) for 20 min at 4°C in the dark. Cells were then washed twice and analyzed on a Fortessa LSR X-20 (BD Biosciences) and analyzed using the FlowJo software v10.10.0 (BD Biosciences). For viral inhibition assays, isolated resting αβ CD4+ T cells from ART-suppressed PWH were directly spinnoculated with HIV_jr-csf_ at 2000 × *g* for 4 hours in the presence of 8 μg/mL polybrene prior to co-culture with effector cells.

### Viral Inhibition assay

Adaptation of the viral inhibition assay was performed as previously reported^34,35^.Briefly, isolated Vδ1 T cells from ART-suppressed PWH were used as effectors and HIV-_jr-csf_ superinfected CD4+ T cells as targets in cocultures at a 1:1 or 1:10 (Effector: Target) ratio. Superinfected CD4+ cells cultured alone were used as a control. Culture supernatants were harvested at day 7 following coculture and stored at −80°C until HIV_p24_ ELISA quantification (ABL, Inc.) was performed. Results are expressed as the percentage of viral inhibition normalized to superinfected CD4+ T cells cultured alone. Additional head-to-head assays were performed to compare viral inhibition from matching Vδ1, Vδ2, αβ CD8 T cells, and NK cells. In half of the head-to-head assays, effectors were pretreated with 25 ng/mL IL-15 (Peprotech) for 24 hours, then washed prior to co-culture with superinfected CD4+ T cells.

### Degranulation and IFN-γ assay

Degranulation assays were performed as previously described^34,35^. Briefly, FACS-isolated Vδ1 T cells were cocultured with either HIV_JR-CSF_-infected autologous CD4+ cells as targets. CD4+ T cells were infected following the approach described above. At least 100,000 effector cells were cocultured at a 1:1 ratio with CD4+ target cells in 96-well plates for 5 hours in the presence of GolgiStop (BD Biosciences) and a CD107a monoclonal antibody (clone H4A3, BD Biosciences). Cells were then harvested, washed with staining buffer, stained with CD3 (clone SK7, Biolegend) and Vδ1 (clone REA117, Miltenyi) for 20 min on ice in the dark, washed twice, resuspended in staining buffer. In parallel cultures, cells were fixed and permeabilized with Cytofix/CytoPerm (BD Biosciences) for 20 min at 4°C in the dark. Cells were then washed in 1X Perm/Wash Buffer (BD Biosciences) and stained for intracellular IFN-γ (clone B27, Biolegend) for 20 min at 4°C in the dark. All samples were acquired in the Attune Acoustic Focusing Cytometer (Applied Biosystems) and analyzed using the FlowJo software v10.10.0 (BD Biosciences).

### Quantitative Viral Outgrowth Assay (QVOA)

QVOA was performed using isolated cell subpopulations from leukapheresis products from ART-suppressed PWH (Table 1) as previously described^8,41^. Briefly, sorted Vδ1 T cells were cultured in limiting dilution from 100,000 to 5,000 cells in replicates of 2 to 9, depending on cell availability and activated with 2.5μg/mL PHA and 100 U/mL IL-2 for 24hours. After washing, isolated and activated CD4+ T cells from uninfected individuals were added to the culture for viral outgrowth and cells were kept in culture for 23 days harvesting the supernatant for HIV_p24_ detection by ELISA (ABL Inc.) at days 15, 19 and 23. Results are shown as Infectious Units per million cells (IUPMs) that represent a maximum-likelihood estimate of the inducible reservoir with 95% confidence intervals^63^.

### Intact Proviral DNA Assay (IPDA)

Genomic DNA was isolated from either FACS-purified Vδ1 T cells or negative magnetic selection (StemCell Technologies) of αβ CD4+ T cells using the QIAmp DNA mini kit (Qiagen). Additional precautions were taken to minimize DNA shearing during isolation including the use of wide-bore pipette tips (Thermo Fisher Scientific) and minimizing the use of vortex mixing. DNA concentrations and purity were measured using a Nanodrop spectrophotometer (Thermo Fisher Scientific). Intact and defective HIV-1 copies/million cells were determined by droplet digital PCR using the IPDA, as previously described^58^. HIV-1 copies were adjusted for shearing and normalized to cell counts based on separate, parallel reactions targeting the *RPP30* gene with previously published primers and probes^57^. Droplets were formed using an Automated Droplet Generator and analyzed on a QX200 Droplet Reader (BioRad) using QuantaSoft software v1.7.4. Each reaction was run in quadruplicate and thresholds for positive signal were determined using both no template and HIV-seronegative controls. Genomic DNA isolated from JLat J89 cells which contain a single integrated copy of HIV-1 was used as a positive control in each assay.

### Next-generation DNAscope in situ hybridization combined to IFA and quantitative image analysis.

We utilized next-generation, ultrasensitive *in situ* hybridization technology for the detection of both HIV DNA (DNAscope) with quantitative image analysis as previously described^40^. We scanned and analyzed full tissue sections (2 sections per sample) to maximize the size of tissue to be assessed. To phenotype the cells harboring vDNA we combined DNAscope with an immunofluorescence approach using antibodies directed to CD4 (polyclonal Goat IgG, 1:500, Abcam), CD8a (polyclonal Rabbit IgG, 1:100, Thremo Fisher Scientific) and/or γδTCR (clone H-41 Mouse IgG, 1:500, Santa Cruz). Slides were washed and then incubated with secondary donkey anti-goat IgG-Alexa Fluor 488, donkey anti-mouse IgG-Alexa Fluor 594 and anti-rabbit IgG-Alexa Fluor 647 (all from Molecular Probes/Thermo Fisher Scientific) for 1 hour at room temperature and washed three times for 5 min in TBS + Tween (0.05% v/v). All slides were counterstained with DAPI (ready-to-use (RTU), Advanced Cell Diagnostics) for 10 min, washed and coverslipped with #1.5 GOLD SEAL cover glass (Electron Microscopy Sciences) using Prolong Gold reagent (Invitrogen). Slides were then scanned using the Akoya Fusion microscope at 40x, and image analysis was performed using QuPath software, v0.4.3^64^. All biopsies were analyzed for up to three tissue sections except for folded or damaged tissue areas. Parameters used for positive cell detection were as follows: background radius 8 μm, median filter radius 0 μm, sigma 1.5 μm, minimum area 10 μm^2^, maximum area 400 μm^2^ and cell expansion 3 μm, and each threshold was adjusted from 15 to 1.5 based on intensity of each marker. Representative pictures were selected to show lamina propria and were extracted using ImageJ.

### Vδ1 T cell activation and cell culture

Cells were either stimulated with 3 μg/mL of plate-bound αCD3 (clone OKT3), 5 μg/mL αCD28 (clone 28.2), and 100 U/mL of IL-2 or left unstimulated for a total of 7 days with half of the media replenished on day 3. Depending on the experiment, cells were harvested at select time points, washed, and stained with a viability dye (Zombie Aqua fixable viability kit, BioLegend) followed by surface staining with monoclonal antibodies (Biolegend) against CD3 (clone SK7), Vδ1 (clone REA117, Miltenyi), CD4 (clone SK3), and CD8 (clone SK1). In some experiments, cells were additionally stained for CD69 (clone FN50), CD25 (clone BC96), HLA-DR (clone L243), CD27 (clone M-T271) CD28 (clone CD28.2), CD45RA (clone HI100), IL-7Rα (clone A019D5), CD62L (clone DREG-56), and CCR7 (clone G043H7). Samples were acquired in a Fortessa LSR X-20 (BD Biosciences) and analyzed using the FlowJo software v10.10.0 (BD Biosciences).

### Proliferation assay

Ten million PBMCs from PWH and HIV-seronegative controls were incubated with 2 μM carboxyfluorescein succinimidyl ester (CFSE) in 1X PBS for 20 min at 37 °C in the dark with continuous shaking. Five times the volume of cRPMI was then added to quench the staining. Cells were then centrifuged at 600 × *g* for 10 min, aspirated, and resuspended at a concentration of 10^6^ cells/mL in cRPMI. Cells were then activated by αCD3/αCD28 + IL-2 stimulation or left unstimulated for a total of 7 days with half of the media replenished on day 3. On day 7, cells were harvested, washed, and stained with a viability dye (Zombie Aqua fixable viability kit, BioLegend) followed by surface staining with monoclonal antibodies against CD3 (clone SK7), Vδ1 (clone REA117), CD4 (clone SK3), and CD8 (clone SK1). Samples were acquired in a Fortessa LSR X-20 (BD Biosciences) and analyzed using the FlowJo software v10.10.0 (BD Biosciences).

### COBRA Assay

Methylation at three potential CpG dinucleotides within the *Cd4* promoter of Vδ1 T cells was determined using Combined Bisulfite Restriction Analysis (COBRA)^65^. Fifty thousand Vδ1 T cells from HIV-seronegative donors were FACS-isolated from stimulated PBMCs (αCD3, αCD28, and IL-2) or unstimulated controls following 5 days in culture. Genomic DNA was directly bisulfite converted using the EZ DNA Methylation-Direct Kit (Zymo Research). Converted DNA was then mixed with 1X PCR Buffer, 1.5 mM MgCl2, 0.2 mM dNTP mix, 0.2 μM Primers, and Platinum Taq (Thermo Fisher Scientific). Primer sequences:

CD4 Bis Forward Primer- GGGGGTGTTAAAGATTATATTTAATTTA

CD4 Bis Reverse Primer- AAACTAAACAAAAAATATCTAAAACACC

Reactions were cycled at 94°C for 2 min, then 35 cycles of (94°C for 30 seconds, 54.5°C for 30 seconds, 72°C for 1 min). Amplicons were then digested with either methylation-sensitive restriction enzyme AccI, HinfI, or Esp3I (New England Biolabs) corresponding to a specific potential CpG site. Digested products were heat-inactivated and directly visualized on a 4% agarose gel with SybrSafe (Thermo Fisher Scientific). Universally non-methylated and methylated human genomic DNA (Zymo Research) were used as negative and positive controls respectively.

### Inhibition of DNA Methylation

Total γδ T cells were isolated from one-hundred million PBMCs from HIV-seronegative donors by negative magnetic selection (StemCell Technologies). Cells were then activated by αCD3/αCD28 + IL-2 stimulation in the presence of 500 nM/mL 5-Azacytidine (5-Aza) or 1X PBS as mock treatment for 5 days. 5-Aza or mock treatment was replenished each day and IL-2 was replenished on day 3 of culture. On day 5, cells were harvested, washed, and stained with a viability dye (Zombie Aqua fixable viability kit, BioLegend) followed by surface staining with monoclonal antibodies against CD3 (clone SK7), Vδ1 (clone REA117), CD4 (clone SK3), and CD8 (clone SK1). Samples were acquired in a Fortessa LSR X-20 (BD Biosciences) and analyzed using the FlowJo software v10.10.0 (BD Biosciences).

### Statistical Analyses

Statistical analyses were performed in GraphPad Prism or with the R programming language. Mann-Whitney U test was used to test for differences between groups whereas Wilcoxon Matched-Pairs Signed-Rank test was used for paired analyses. Significant correlations between Vδ1 T cell phenotypes, HIV-1 DNA, and participant characteristics were calculated with Spearman’s ranked correlation test. Two-way ANOVA was used to determine differences between multiple groups over time. Technical replicates were averaged, and outliers were excluded from analysis when greater than ± 2 s.d from the mean.

## Extended Data

**Extended data Figure 8:**
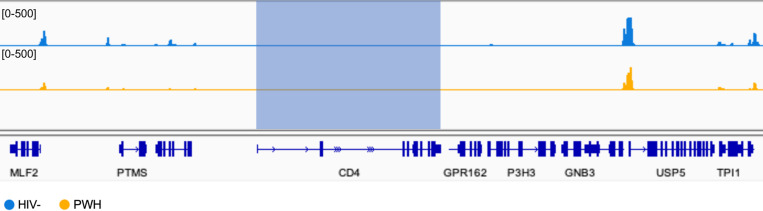
Chromatin Accessibility of *Cd4* locus. Integrated genome viewer window showing reduced ATAC-seq peaks (highlighted in blue) within the *Cd4* of both HIV- donors (blue) and ART-suppressed PWH (orange).

**Extended data Figure 9:**
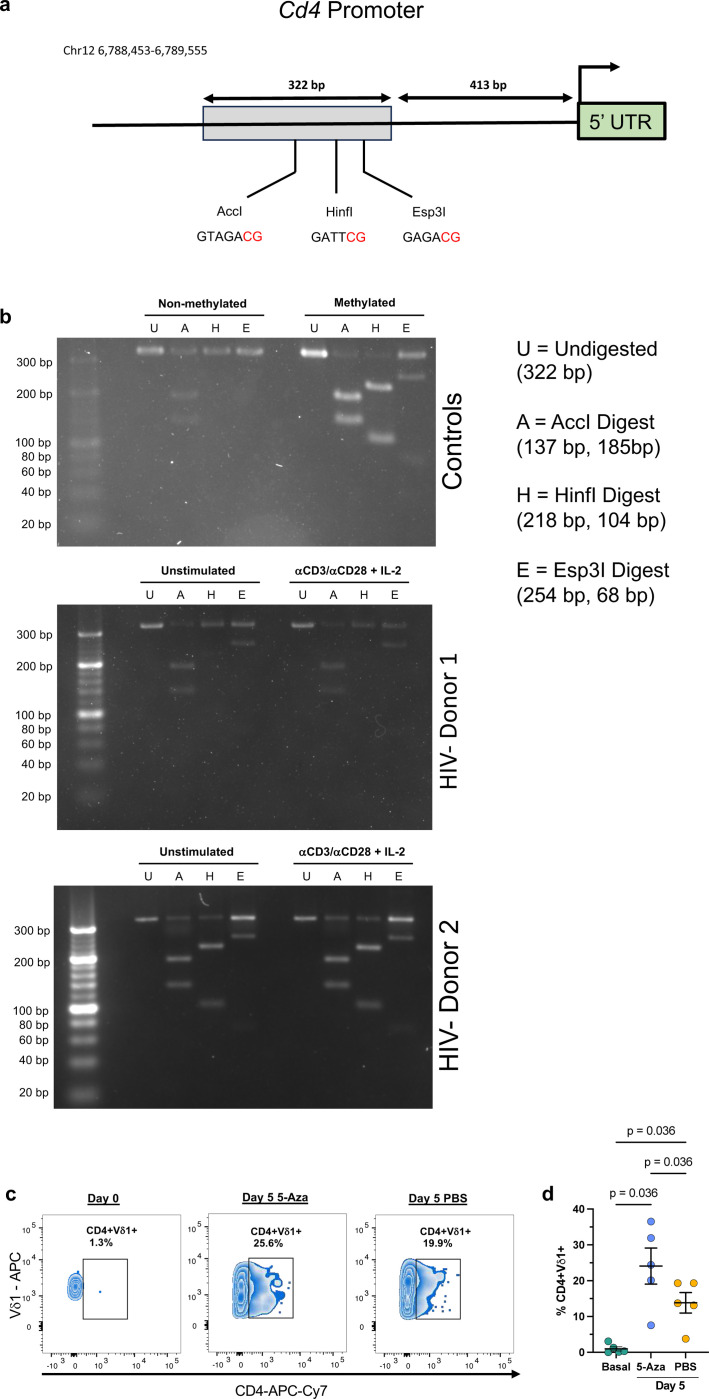
Detection of CpG methylation within the *Cd4* promoter region of Vδ1 T cells. **a**, Schematic representation of the *Cd4* promoter with the grey box outlining the 322 bp region where DNA methylation was assessed in Vδ1 T cells using combined bisulfite restriction analysis (COBRA). The assay utilized three methylation-sensitive endonucleases: AccI, HinfI, and EspI with specific cut sites corresponding to three separate potential CpG dinucleotides (red) within the promoter. **b**, Top: Representative COBRA assay showing genomic DNA from universally non-methylated and methylated controls either undigested (U), AccI digested (A), HinfI digested (H), or Esp3I digested (E). The universally methylated control shows the expected band sizes for each digest (shown on right). Middle and Bottom: COBRA assay of purified Vδ1 T cells from two separate HIV- donors either unstimulated or stimulated with αCD3/αCD28 + IL-2 for 5 days. **c**, Representative zebra plots and **d**, frequency of CD4+Vδ1 T cells from HIV- donors (n = 4) at day 0 and day 5 after stimulation with αCD3/αCD28 + IL-2 and daily treatment with either 5-Azacytidine (5-Aza) or PBS. One-way repeated measures ANOVA and Holm-Šidák method for multiple comparisons (**d**). Mean ± S.E.M. represented.

**Extended data Figure 10:**
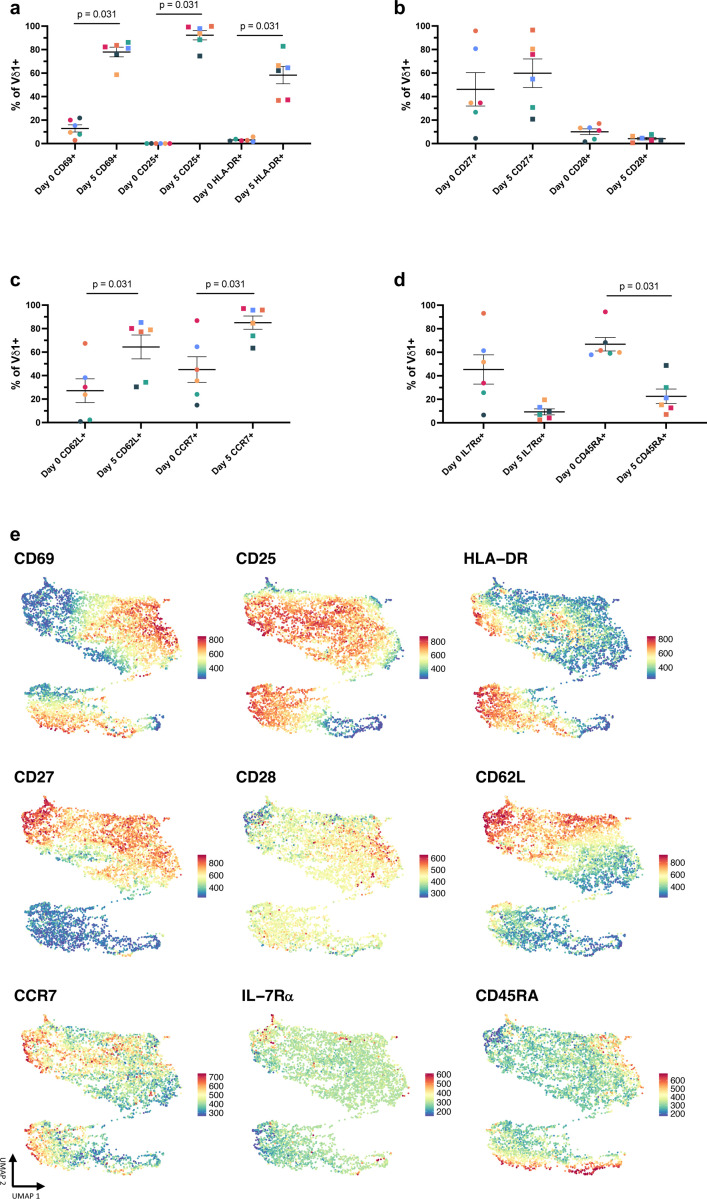
Co-expression of CD4 with markers of activation and memory. Comparison of **a**, activation markers, **b**, co-stimulatory receptors, **c**, lymphoid homing molecules, and naïve T cell markers on Vδ1 T cells at day 0 and day 5 following stimulation of PBMCs from HIV-donors with αCD3/αCD28 + IL-2. **e**, Heatmap representation of the relative expression of each marker overlayed with UMAP displaying clusters identified by FlowSOM analysis (Fig 6k-m) of day 5 cultures following stimulation (n = 6). Wilcoxon matched pairs signed rank test.(**a-d**). Mean ± S.E.M represented.

## Figures and Tables

**Table 1.** Participant characteristics

	ART PWH GW/UNC	ART PWH SV	EC SV
N	46	4	3
Median Age (years) (IQR)	36 (30–42)	40 (32–46)	52(51–54)
**Sex**			
Male (%)	42 (91.3%)	4(100%)	2(66.6%)
Female (%)	4 (8.7%)		1(33.3%)
**Race (%)**			
African-American	20 (43.5%)		
Non-Hispanic, White	19(41.3%)	4(100%)	3(100%)
Hispanic, White	3 (6.5%)		
Asian	2 (4.3%)		
Other/Unknown	2 (4.3%)		
**Median time since diagnosis (months) (IQR)**	**89 (41–156)**	**92(81–103)**	**310(290–343)**
Median CD4 nadir (cell/mm^^^3) (IQR)	420(324–531)	499 (423–594)	433 (313–523)
**Stage at time of ART initiation) (%)**			
Acute (%)	11 (23.9%)		
Chronic (%)	35 (76.1%)	4(100%)	
**Median time on ART (months) (IQR)**	**72 (37–113)**	**60 (47–85)**	
Median time suppressed (<50 copies/ml) (months) (IQR)	44(27–91)	56 (38–80)	
Median CD4 count (cell/mm^^^3) (IQR)	719(615–851)	1103 (984–1143)	758 (552–782)
